# Consideration of the Intricacies Inherent in Molecular Beam Epitaxy of the NaCl/GaAs System

**DOI:** 10.1021/acsomega.2c00954

**Published:** 2022-07-01

**Authors:** Brelon J. May, Jae Jin Kim, Patrick Walker, William E. McMahon, Helio R. Moutinho, Aaron J. Ptak, David L. Young

**Affiliations:** † National Renewable Energy Laboratory, Golden, Colorado 80401, United States; ‡ Shell International Exploration and Production, Inc., Houston, Texas 77079, United States

## Abstract

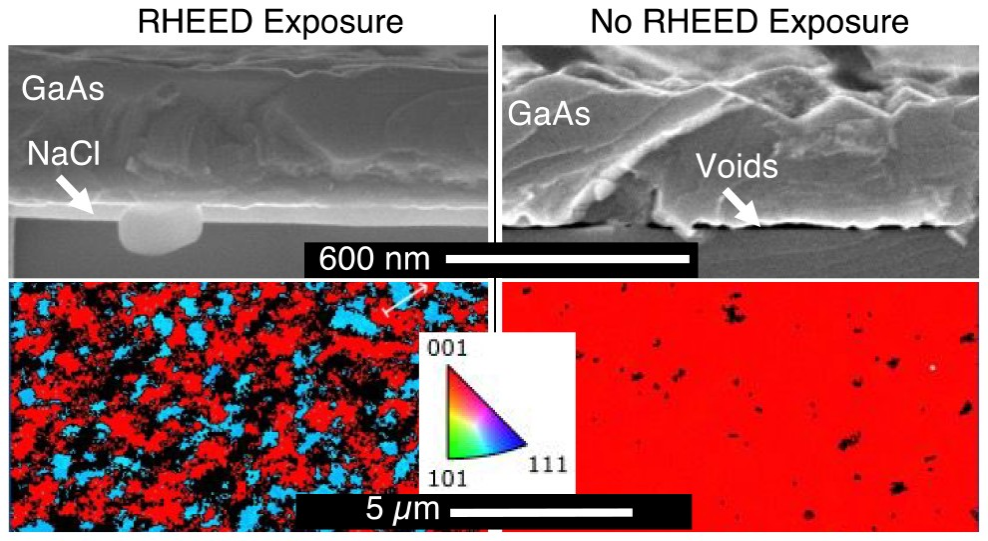

The high cost of substrates for III–V growth can be cost limiting for technologies that require large semiconductor areas. Thus, being able to separate device layers and reuse the original substrate is highly desirable, but existing techniques to lift a film from a substrate have substantial drawbacks. This work discusses some of the complexities with the growth of a water-soluble, alkali halide salt thin film between a III–V substrate and overlayer. Much of the difficulty stems from the growth of GaAs on an actively decomposing NaCl surface at elevated temperatures. Interestingly, the presence of an *in situ* electron beam incident on the NaCl surface, prior to and during GaAs deposition, affects the crystallinity and morphology of the III–V overlayer. Here, we investigate a wide range of growth temperatures and the timing of the impinging flux of both elemental sources and high energy electrons at different points during the growth. We show that an assortment of morphologies (discrete islands, porous material, and fully dense layers with sharp interfaces) and crystallinity (amorphous, crystalline, and highly textured) occur depending on the specific growth conditions, driven largely by changes in GaAs nucleation which is greatly affected by the presence of the reflection high energy electron diffraction beam.

## 1 Introduction

Substrate recycling is an area of high interest, especially for technologies that employ expensive single-crystalline materials such as epitaxial III–Vs. The cost of substrates is still problematic for technologies requiring a large area such as high-efficiency solar cells,^1^ while processing costs can be reduced through scaling and growth costs are being reduced with technologies such as high growth rate metal organic chemical vapor deposition (MOCVD)^2,3^ and hydride vapor phase epitaxy (HVPE).^4−6^ A number of methods have been demonstrated for separation of a III–V device layer from the parent wafer that can be thought of as either spalling (mechanical removal)^7−9^ or wet etching of a sacrificial layer (chemical removal).^10,11^ Both techniques have advantages and disadvantages pertaining to the material(s) used, the fracture planes, and the substrate orientation used. It is paramount that the surface remains as pristine as possible to obtain the benefit of cost effectiveness, and existing methods on commonly used GaAs (001) substrates fall short. For spalling, there is a mismatch in the desired crack propagation direction and the low-energy cleavage planes resulting in facets at a high angle to the surface normal when spalling GaAs (001) substrates. Existing sacrificial layers for wet-etching techniques (Al-rich III–Vs) also tend to leave behind a rougher surface and insoluble byproducts on the surface.^12^ This work explores the integration of NaCl as a different selective etch layer material in an effort to preserve the epi-ready surface of the parent wafer post exfoliation.

The high solubility of NaCl and low solubility of III–Vs in water presents an alternative avenue for the possibility of a rapidly dissolvable sacrificial layer with high selectivity in a nontoxic environment and was shown to be effective in various material systems and devices.^13−15^ Both GaAs and NaCl are cubic, and while NaCl has a higher thermal expansion coefficient, they are lattice matched at ∼100 °C. The first integration of these two materials occurred in the early days of epitaxy and vacuum deposition, with NaCl being a popular substrate choice for both semiconductors and metals.^16−23^ Previous demonstrations of growth of GaAs on NaCl used bulk NaCl substrates that suffered from reactivity with water vapor in the air. These substrates were vacuum-cleaved and deliberately desorbed large amounts of material *in situ* prior to growth to produce a clean surface.^24^ However, bulk NaCl substrates are likely not economical for device exfoliation because the substrate would necessarily be dissolved after each growth. A more elegant approach is to deposit an epitaxial NaCl liftoff layer directly on a GaAs substrate to facilitate liftoff and substrate reuse. This requires the development of the growth of both NaCl on GaAs and GaAs on NaCl. This is not a trivial combination because of the possibility of forming antiphase domains and twin boundaries when growing GaAs on NaCl because of the higher symmetry of NaCl. The most challenging obstacle is that NaCl is highly volatile at typical GaAs growth temperatures approaching 600 °C. Thin NaCl layers can completely (or partially) desorb at much lower temperatures prior to becoming fully encapsulated by the GaAs. The direct growth of a monocrystalline GaAs layer directly on a NaCl thin film was only demonstrated recently by our group by using very short alternating pulses of Ga and As to promote adatom mobility and better surface coverage with thinner layers.^25^ The work discussed here will present the intricacies with the deposition of NaCl, subsequent GaAs deposition directly on rapidly desorbing NaCl thin films at elevated temperatures up to 500 °C, and the convoluted interplay with exposure of the NaCl layer to a reflection high energy electron diffraction (RHEED) electron beam which is the foundation that led to the successful demonstration of single-crystal GaAs on NaCl.

## 2 Results and Discussion

An array of intertwining results will be presented in the following section. First, the temperature dependence of the deposition of NaCl thin films on GaAs substrates will be presented in section 2.1. The NaCl layer is crystalline and oriented parallel to the substrate with compositionally abrupt interfaces. With thin films of NaCl being reproducible, section 2.2 pertains to work done on the growth of GaAs on these thin NaCl layers with a large focus on the substrate temperature. Initializing the GaAs growth at low temperature (<300 °C) yields poor crystallinity of GaAs on NaCl thin films. However, NaCl begins to decompose rapidly above 300 °C; withholding deposition of the GaAs overlayer until temperatures >500 °C result in complete desorption of the NaCl layer and homoepitaxial GaAs layers. The NaCl must be capped rapidly at lower temperatures to combat excessive desorption of the NaCl at elevated temperatures. However, GaAs growth on NaCl proceeds via the formation of discrete islands, and the relatively thick layers that must be grown at low temperatures in order to coalesce largely spaced islands without desorption of the NaCl result in poor crystallinity.

Section 2.3 shows how exposure of the NaCl surface to the RHEED beam can positively affect the nucleation of GaAs. We show that the presence of the RHEED beam during and even prior to GaAs deposition influences the morphology of the overlayer. Large changes were observed between areas exposed to RHEED during the GaAs deposition; a scheme was developed wherein the NaCl surface was exposed to the RHEED beam under an As flux at low temperature prior to the GaAs deposition. Under these conditions, an amorphous As layer would condense on the NaCl surface only where the RHEED beam was exposed, and the entire sample could be covered relatively uniformly. The amorphous layer desorbs from the surface upon heating to the temperature where GaAs is subsequently grown. The GaAs nucleates more rapidly and better protects the NaCl in these RHEED exposed areas. To best utilize this effect, the GaAs growth was eventually separated into a nucleation step where a thin (<100 nm) GaAs layer is deposited at low temperature and then heated to 580 °C for further GaAs growth at more typical conditions. This procedure resulted in highly textured GaAs on complete NaCl thin films after continued growth at 580 °C and is the basis of additional work outside the scope of this paper which yielded monocrystalline GaAs.

### 2.1 Deposition of NaCl Thin Films on GaAs (001) Substrates

**Figure 1:**
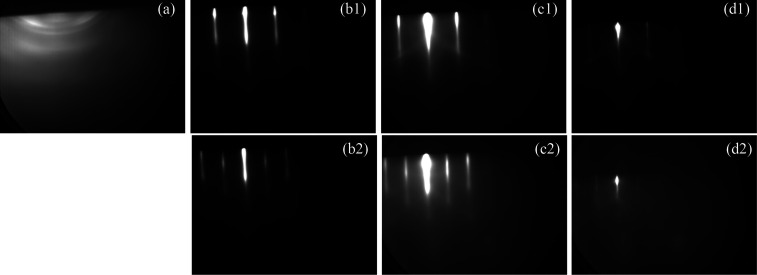
RHEED images of different samples with 10 min (roughly 30 nm) of NaCl deposition on (a) a GaAs substrate at 100 °C with a small amount of excess As on the surface and on clean GaAs surfaces at (b) 100 °C, (c) 150 °C, and (d) 175 °C. Top row (1) taken along the [100]. Bottom row (2) taken along [110].

The first step is to understand the growth of crystalline NaCl on GaAs (001) substrates. First, a 300 nm GaAs buffer layer is grown at 580 °C; after completion, RHEED reveals the typical As-terminated 2 × 4 surface reconstruction. The diffraction pattern converts to a symmetric c(4 × 4) as the sample is then cooled under an As overpressure (∼1 × 10^–6^ Torr) until ∼340 °C. The sample is cooled to the desired temperature for the growth of NaCl (*T*_NaCl_). A nominally 30 nm thick layer of NaCl is deposited using a NaCl deposition time (*t*_NaCl_) of 10 min and a growth rate of ∼3 nm/min. If the As source remains open when the substrate temperature is <320 °C, the RHEED pattern begins to go diffuse as a result of the condensation of small amounts of amorphous As. In this case, a ringlike pattern quickly appears upon opening of the NaCl shutter which does not revert back to a streaky single-crystalline pattern with further NaCl deposition (Figure 1a1). Blurry arcs superimposed on the rings signal some degree of fiber texturing,^26^ which in the absence of a crystalline surface on which to nucleate would suggest that NaCl has a preferred growth direction.

The remaining images of Figure 1 show RHEED patterns viewed along the [110] and [100] directions of 30 nm of NaCl deposited at various *T*_NaCl_ starting on clean c(4 × 4) GaAs surfaces and a chamber pressure less than <7 × 10^–8^ Torr. Deposition at 100 °C (Figure 1b) is most closely lattice matched with GaAs and displays a streaky pattern with slight undulations indicating small island growth.^27^Figure 1c shows patterns from deposition of NaCl at 150 °C. The streaks become brighter and Kikuchi patterns become visible, indicating that the surface becomes increasingly well-organized and smooth despite a slight increase in the lattice mismatch (which increases from ∼0% at 100 °C to 2.9% at 580 °C) due to the difference in thermal expansion between the materials. Figure 1d shows that deposition of NaCl at 175 °C results in a dimmer pattern overall which could be for two reasons which will be discussed more in sections 2.2.2 and 2.3. First, the exposure of the NaCl to the RHEED beam begins to have substantial effects on the salt layer at higher temperatures. Second, as the substrate temperature is increased, the NaCl layer begins to decompose. Thus, the nucleation of NaCl at temperatures >175 °C was not studied. When deposition occurs on clean GaAs surfaces (Figures 1b–d) the ratio of the spacing between streaks along the [110] and [100] directions is proportional to √2/2. Additionally, the [110] and [100] patterns of the NaCl are parallel to the [110] and [100] directions of the GaAs, indicating that the NaCl has a cubic symmetric crystalline surface.

**Figure 2:**
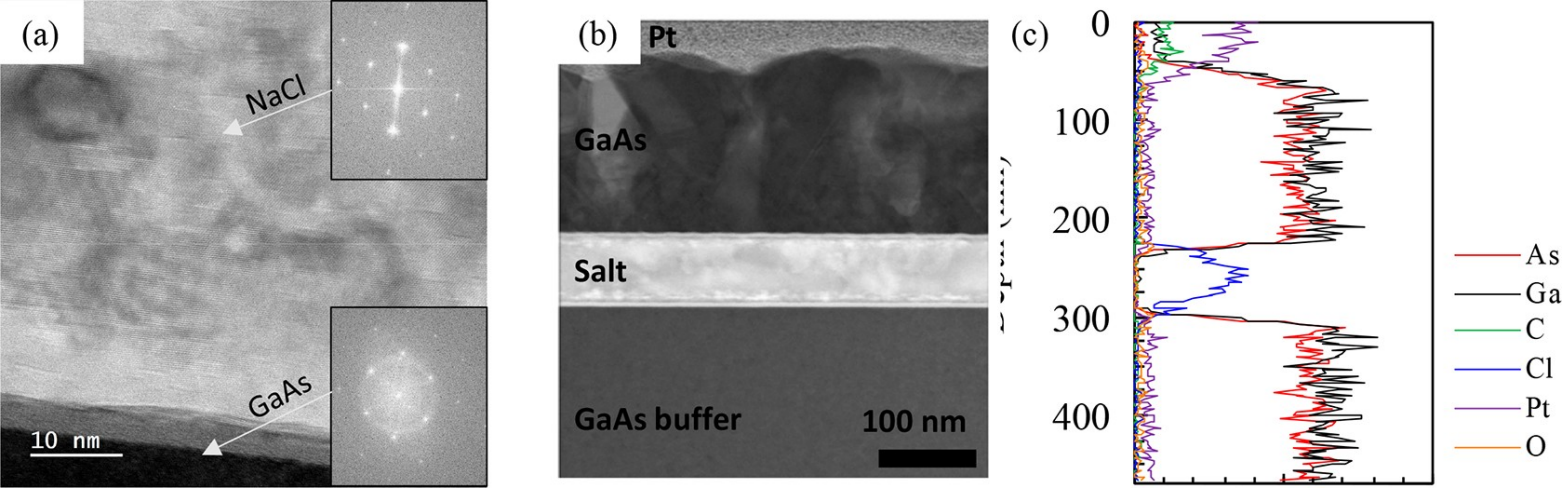
(a) STEM image of the NaCl layer with insets showing FFTs of the (top) NaCl layer and (bottom) GaAs interfacial area. (b) STEM image of a NaCl layer capped with GaAs. (c) EDX maps of the NaCl layer contained between GaAs layers. (d) Line scans for each element.

Further insight into the crystallinity, structure, and diffusion of the NaCl layers was desired. Thus, 90 nm NaCl (deposited at 150 °C) was capped with GaAs at 350–580 °C (details on the GaAs deposition procedures will be discussed in sections 2.2 and 2.3). Figure 2a shows a scanning transmission electron microscopy (STEM) image with fast Fourier transforms (FFTs) of both the NaCl and GaAs substrate. The FFTs reveal that the NaCl layer is oriented the same as the substrate and has nearly identical lattice constants. Organized atomic planes of NaCl are observed, but they are not perfectly straight. Contrast variations in the NaCl layer are artifacts resulting from damage induced by the electron beam during acquisition (further discussed in section 2.3 and the Supporting Information S1), which could be a reason for the tilting of some of the atomic planes. There is also a dark ∼5 nm layer at the interface between the GaAs substrate and NaCl film. This layer appears amorphous, but the rings in the FFT of the GaAs area suggest that it might be nanocrystalline. It is possible that this is due to beam damage, but a separate investigation of the NaCl deposition on different GaAs surface reconstructions revealed some degree of textured polycrystalline growth during the early moments of deposition.^25^ However, an organized (001) surface is achieved by the end of the NaCl deposition, as shown previously in Figure 1.

Parts b and c of Figure 2 shows a lower magnification STEM image and the corresponding energy-dispersive X-ray spectroscopy (EDX) line profile. The image reveals the presence of a similar upper GaAs/NaCl interface, but the EDX shows that the interface with the substrate is compositionally sharper than this upper interface. There is no Ga or As observed in the bulk of the NaCl layer. Additionally, the Cl is also well contained in the NaCl and no appreciable outward diffusion is seen. The signal for Na is not plotted here because the main K-peak overlaps the dominant Ga L-peak; the Ga signal is taken using the K-family peaks to avoid contamination from any Na signal. Thus, the Cl data combined with the STEM is used to verify the presence of a NaCl layer and is only present within the bright layer. Secondary ion mass spectroscopy (SIMS) profiles of additional samples (not shown) with NaCl films capped with GaAs reveal that exposure to high temperature (620 °C) may cause Na diffusion into the surrounding GaAs. However, the Na diffusion area can be outgrown, with the concentration returning to the same level as the underlying substrate within a few hundred nanometers. A slight increase in oxygen levels at both interfaces is observed, which is likely due to the growth pauses between the buffer layer and the NaCl and the NaCl and the GaAs cap. This could also be related to the interfacial layers at both interfaces observed in the STEM image. The only presence of C is in the protective layer deposited during sample preparation, as also marked by the Pt signal.

### 2.2 Deposition of GaAs on NaCl Thin Films

#### 2.2.1 Temperature Dependence of Initial GaAs Nucleation

**Figure 3:**
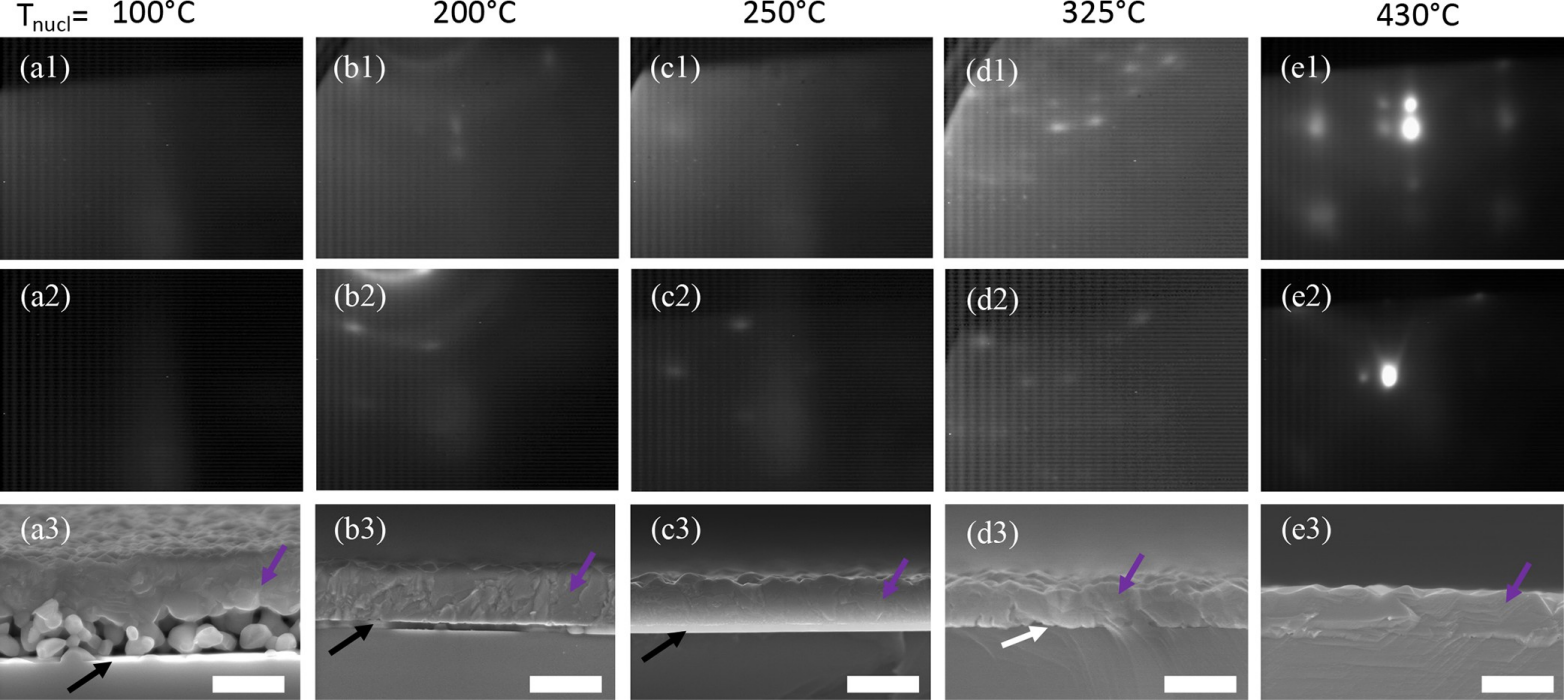
RHEED images taken with a ⟨100⟩ beam direction during (1) the initial and (2) the final moments of GaAs deposition and (3) cross-sectional SEM of the finished sample (scale bar is 600 nm) after 30 min of salt deposition capped with GaAs initially at a temperature of (a) 100 °C, (b) 200 °C, (c) 250 °C, (d) 325 °C, and (e) 430 °C and ramped to 580 °C. Purple, black, and white arrows mark the GaAs overlayer, an NaCl layer, and any voids between the substrate and overlayer, respectively.

The subsequent deposition of GaAs layers on NaCl thin films was carried out in the same molecular beam epitaxy (MBE) chamber without any vacuum break. The first investigation involves varying the initial GaAs nucleation temperature (*T*_GaAs1_) for GaAs deposition on a NaCl layer. A schematic of this growth process is given in the Supporting Information S2(a). After growth of the high-temperature buffer layer, nominally 90 nm of NaCl is deposited at 110 °C. The NaCl shutter is closed, and the temperature is increased at a rate of 50 °C/min to the nucleation temperature under test. GaAs deposition begins once *T*_GaAs1_ is reached at a rate of ∼33 nm/min (calibrated using RHEED oscillations during homoepitaxial GaAs growth) by simultaneously opening both the Ga and As sources while the substrate temperature is continuously increased to 580 °C. The resulting thicknesses of each sample are slightly different because the total GaAs deposition time (*t*_GaAs1_) depends on the nucleation temperature and the ramp rate (samples with lower *T*_GaAs1_ are thicker). Figure 3 shows RHEED patterns at the onset and at the end of GaAs deposition with corresponding cross section SEM images from a series of growths with various *T*_GaAs1_. The additional spots in the RHEED patterns located to the left of the primary and first order spots here are artifacts of the incident RHEED beam and not indicative of any surface reconstructions or twin grain structure.

When GaAs deposition begins at 100 °C (Figure 3a) the RHEED pattern goes diffuse very quickly, signifying a lack of crystalline order. Due to the increased surface roughness and spontaneous delamination of the film from the substrate, the pattern gets darker upon continued growth and heating, as even diffuse reflections are blocked. In contrast to the other samples in this figure, the sample in Figure 3a has an additional ∼90 nm of GaAs at *T*_GaAs1_ (110 °C) prior to heating to 580 °C. Without this longer initial low-temperature deposition the film completely delaminated from the substrate during the growth. Cross sectional SEM reveals an extremely porous interface with a coalesced top layer. Additional *ex situ* STEM measurements (not shown) reveal the smaller particles between the substrate and film to be crystalline GaAs, with the overlayer being a dense polycrystalline film with grains on the order of 100 nm. Additionally, if the temperature is not increased from 110 °C, the film remains smooth but fully amorphous and As rich (Supporting Information S3a). Additional samples (not shown) showed that changing the thickness of the initial 110 °C deposition results in proportional changes in the thickness of the porous section. However, the coalesced top region remains similar in thickness and without any observed improvements in the crystallinity. Thus, it can be assumed that the porous structure is a result of the low-temperature, As-rich, amorphous deposition, and the coalesced layer is due to the growth at elevated temperatures.

The formation of a porous interface can be avoided by increasing the *T*_GaAs1_ to 200–250 °C (Figures 3b,c). In both cases, the RHEED develops a spotty ring pattern upon initial GaAs deposition, which persists throughout growth indicating a textured polycrystalline film. The presence and persistence of the polycrystalline rings decreases as *T*_GaAs1_ is increased. SEM shows the presence of a smooth ∼70 nm thick NaCl layer maintained beneath a fully dense ∼500 nm GaAs overlayer. STEM and electron backscatter diffraction (EBSD) measurements reveal that these films are indeed polycrystalline, in agreement with the RHEED observations.

If the NaCl film is heated to temperatures ≥325 °C prior to initiating the GaAs deposition (Figure 3d,e the RHEED immediately begins to turn spotty, indicating Volmer–Weber style growth. Little change is observed throughout the deposition at 325 °C. However, using *T*_GaAs1_ = 430 °C, the pattern at the end of the GaAs deposition shows spots with chevrons indicating shallow surface facets. In either case with *T*_GaAs1_ ≥ 325 °C, SEM measurements no longer show the presence of a NaCl layer. Instead, large gaps between the substrate and a rough GaAs surface layer are observed. As the *T*_GaAs1_ is increased, the large gaps become smaller pores and eventually disappear altogether. The NaCl film begins to rapidly decompose as the sample is heated above 300 °C; as a point of reference the NaCl effusion cell temperature is operating ∼480 °C.

The observation of the initial spotty RHEED in each scenario indicates that initial GaAs on NaCl growth proceeds via three-dimensional island growth. This results in incomplete coverage of the NaCl until all islands coalesce. This enables the NaCl to continuously desorb at the higher temperatures even during GaAs deposition. This happens especially quickly at higher temperatures where both the nucleation of islands is slower and the NaCl desorbs more rapidly. For example, at 300 °C some GaAs is seeded on the NaCl before desorbing, but not quickly enough to form a cohesive film before the NaCl is completely desorbed, leaving behind large voids. By delaying GaAs deposition until 430 °C, most of the NaCl has already desorbed from the surface. It is likely that by this point the NaCl layer is either very thin or has completely disappeared, and many of the initial GaAs atoms are impinging directly on a GaAs surface, resulting in homoepitaxy. Any remaining NaCl escapes through pinholes or gaps between GaAs islands, and the result is small pores in a mostly homoepitaxial structure. Additional X-ray diffraction (XRD) and STEM measurements (not shown) confirm the RHEED measurements that the GaAs overlayers of any previously discussed case, with a persistent salt layer or large voids (*T*_GaAs1_ ≤ 325 °C), are polycrystalline. Additionally, using a *T*_GaAs1_ = 580 °C, the NaCl is completely desorbed prior to GaAs nucleation. In this case, the RHEED remains very streaky and regains the typical reconstructions observed with MBE growth, and cross-sectional SEM shows no evidence that a NaCl layer was ever deposited. Intriguingly, this suggests that any NaCl can be thermally cleaned and regrowth on substrates which once had salt deposited on them is possible.

#### 2.2.2 Persistent NaCl Layers at Elevated Temperatures

Investigation of GaAs deposited on NaCl at temperatures >300 °C requires accounting for NaCl desorption upon heating. A new growth scheme was tested to combat this extra desorption where the temperature is now also held steady for the entirety of GaAs deposition (*t*_GaAs1_ = 9 min) instead of being continuously ramped. An initial NaCl layer is deposited at 110 °C (*t*_NaCl_ up to 180 min) and is continuously deposited while increasing the temperature to *T*_GaAs1_ at a rate of 20 °C/min (outlined in the Supporting Information S2b). The time it takes to ramp to this temperature (*t*_ramp1_) changes with the *T*_GaAs1_ chosen. The highest temperature case (*T*_GaAs1_ = 500 °C) has a *t*_ramp1_ ∼ 19.5 min, for example. Additionally, the growth rate was increased up to ∼17 nm/min. Thus, neglecting any desorption and accounting for the NaCl deposited during both *t*_ramp1_ and *t*_NaCl_ (19.5 and 180 min), the thickest NaCl layers deposited were ∼3.3 μm. RHEED images of the NaCl surface during deposition and heating to the growth temperature are shown and discussed in Supporting Information S3.

**Figure 4:**
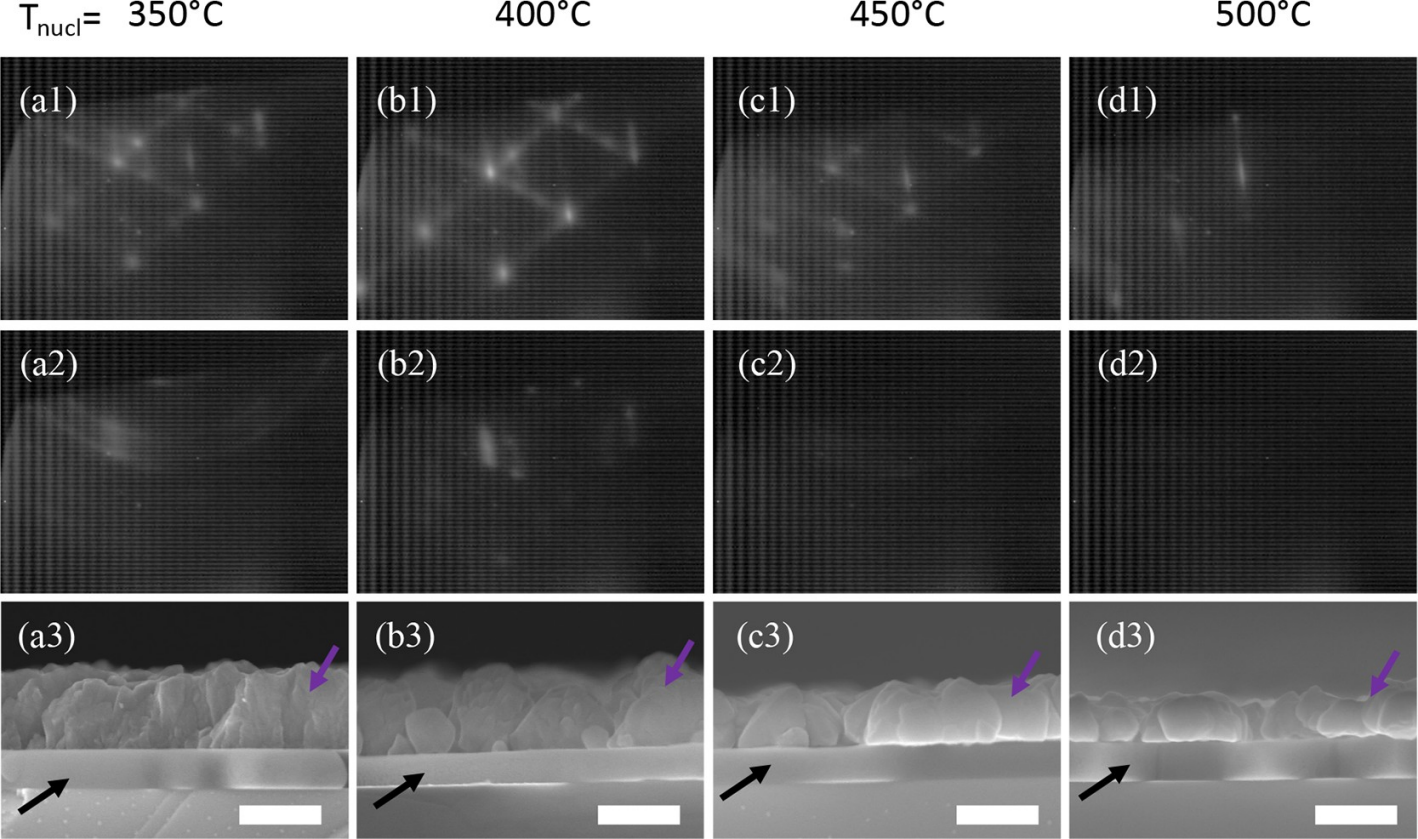
RHEED images of (1) initial and (2) final GaAs deposition on thick NaCl and (3) the corresponding SEM images of samples with NaCl deposition beginning at 110 °C and continuously until the GaAs cap is initialized at (a) 350 °C, (b) 400 °C, (c) 450 °C, and (d) 500 °C. Purple and black arrows mark the GaAs overlayer and NaCl layer, respectively (scale bars are 300 nm).

RHEED and cross-section SEM patterns are used to analyze the growth of GaAs on the thick continuously deposited NaCl films. Parts a1–d1 of Figure 4 show the RHEED patterns during the initial moments of GaAs deposition at various *T*_GaAs1_. Samples with *T*_GaAs1_ ≤ 450 °C show complex RHEED patterns with symmetric shadow spots and chevrons within the first ∼10 s of deposition. The extra set of spots symmetric about the primary reflections are expected to be due to twins, likely rotations about the {111} which will be discussed more in section 2.3.2. The shallow angle chevrons as viewed along this ⟨110⟩ direction correspond to {111} faceting of the GaAs. However, at *T*_GaAs1_ = 500 °C the RHEED during initial growth remained streaky and it took nearly 60 s to display a RHEED pattern similar to what was observed at the lower temperatures (Figure 4d1). In this case, it is possible that the initial GaAs islands are more epitaxially oriented and have a lower degree of twin formation. However, it is more likely that the longer time before a similar pattern is observed is due to a slower nucleation and growth of GaAs at this elevated temperature.

The middle row of Figure 4 shows the RHEED patterns at the end of the GaAs deposition at the different temperatures. For *T*_GaAs1_ = 350 °C (Figure 4a2), the pattern at the end of the ∼300 nm deposition is spotted and ringlike, indicating a textured polycrystalline film, in contrast to the original pattern. Figure 4b2 also shows a spotty ring pattern for the growth at *T*_GaAs1_ = 400 °C. The rings are comparatively less pronounced than the spots, but the pattern is dimmer overall. This indicates a higher degree of crystalline order compared to growth at lower temperature but still a very rough film. It was impossible to discern a pattern at the end of the growth for depositions at *T*_GaAs1_ ≥ 450 °C. The pattern become steadily darker as the growth temperature is increased, likely due to the formation of increasingly discrete islands. Electrons at the RHEED energies have a mean free path on the order of a few tens of nanometers. Thus, these glancing-angle electrons are completely blocked by the islands which are hundreds of nanometers in size, and the pattern on the phosphor screen becomes dark.

The bottom row of Figure 4 shows the corresponding SEM images of the previously discussed samples. All samples have the same *t*_GaAs1_ and growth rate (33 nm/min) and thus should have the same target thickness of 300 nm. Figure 4a3 shows that GaAs deposited at 350 °C is dense and approximately equal to the target thickness. As *T*_GaAs1_ increases, the morphology trends toward the formation of more discrete faceted islands, in agreement with the RHEED observations from the early portions of the growth. Additionally, as the *T*_GaAs1_ goes higher than 450 °C the islands become smaller and more discrete. This also corresponds with the darkening of the RHEED pattern at these temperatures because an array of disconnected ∼200 nm tall islands is essentially a very rough film. Figure 4d3 shows that with *T*_GaAs1_ = 500 °C the islands are thinnest (∼190 nm), only ∼60% of the targeted thickness. The reason behind this reduction in thickness, or apparent growth rate, with increasing temperature is not fully understood. It is possible that the rapid desorption of the NaCl surface at these higher temperatures creates chemical complexes, such as GaCl_3_, which are more volatile and halt further growth or that impinging Ga and As atoms have difficulty remaining on such an actively desorbing surface because the temperature of the substrate temperature is now higher than the NaCl effusion cell.

**Figure 5:**
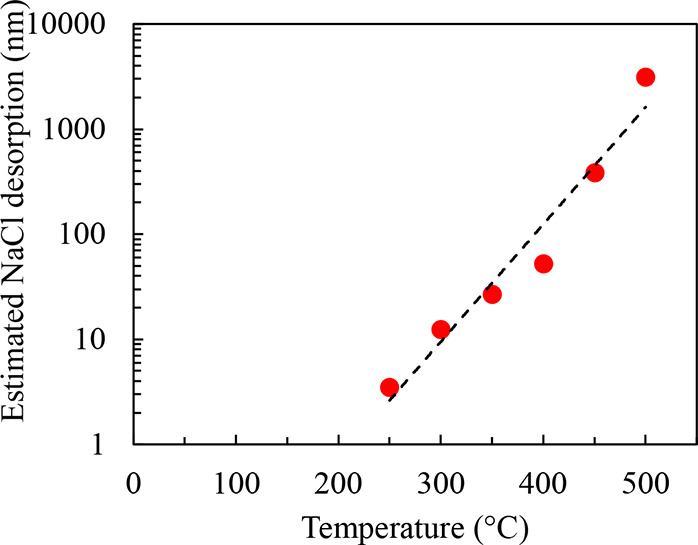
Amount of salt desorption as a function of GaAs capping temperature.

Despite the NaCl thicknesses all appearing similar in these images, there are significant differences between the total amount of NaCl deposited. While the estimated and observed thicknesses of NaCl are similar when *T*_GaAs1_ ≤ 250 °C (Supporting Information S4), they begin to strongly diverge as the temperature increases. Figure 5 shows the estimated amount of total NaCl desorption using the difference between the expected thickness (from measured low temperature growth rates and total NaCl deposition time) less the thickness observed in the SEM images from Figure 4. As mentioned earlier, the thickest NaCl layer was deposited for *T*_GaAs1_ = 500 °C (∼3.3 μm), and the observed thickness is only ∼160 nm. Thus, >3 μm of material had desorbed during the growth of this sample. The initial NaCl thickness required to maintain a persistent film until the end of growth increases exponentially as the growth temperature is increased. The actual desorption rate of the NaCl is temperature dependent, but lower and upper bounds on the desorption rate can be made by making two generous assumptions using the sample with *T*_GaAs1_ = 500 °C: the NaCl decomposition either occurs (1) over the entire 29 min at which the sample is >110 °C or (2) exclusively during the 9 min growth at 500 °C. These would result in an average desorption rate somewhere in the range of 112–362 nm/min. This desorption rate would be in the range of 3.4–11× the growth rate of GaAs used in this study. This presents an obvious challenge for achieving growth at typical GaAs deposition temperatures which are even higher.

### 2.3 The Effect of RHEED on GaAs/NaCl Growth

#### 2.3.1 Effect of RHEED Exposure Prior to and during GaAs Deposition on NaCl

As demonstrated throughout the earlier sections, RHEED is a critical tool for *in situ* observation, but the presence of the electron beam during growth actively affects the growth surface, which has been seen in other material systems.^28,29^ In an attempt to elucidate the effects of the presence of the electron beam, a sample was grown where the beam was moved across the surface at different points during the growth, resulting in different levels of exposure and exposure starting at different times during the growth process. There is an obvious difference between regions with and without beam exposure even by eye. Images of the marks left by the 15 kV RHEED beam on this sample and discussion of the RHEED patterns from the growth are contained in Supporting Information S5. The growth process used here was similar to that discussed in section 2.2.2, i.e., 10 min of initial NaCl deposition at 110 °C followed by continuous deposition of NaCl while heating to 300 °C, at which point GaAs is deposited at a rate of 33 nm/min to reach a nominal thickness of 100 nm.

**Figure 6:**
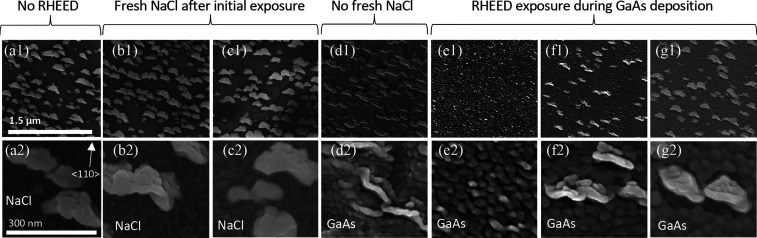
Plan view SEM images of 100 nm of GaAs deposited on a NaCl film at 300 °C at (1, top row) low and (2, bottom row) high magnification. (a) Not exposed to RHEED at any point during the growth. (b) Exposed to RHEED during NaCl deposition and covered with >20 nm of NaCl without RHEED exposure. (c) NaCl exposed to RHEED for 180 s and covered with 4.5 nm of NaCl without exposure. (d) NaCl exposed to RHEED for 90 s immediately prior to GaAs deposition. RHEED exposed to the area during the (e) initial, (f) second, and (g) third minute of GaAs deposition.

Figure 6 shows plan-view SEM images of seven distinct areas on the same sample with various degrees of RHEED exposure (RE) at different times throughout the growth at low and high magnification, top and bottom rows, respectively. Details, RHEED patterns, and a schematic of the RHEED exposure during the growth process are further discussed in Supporting Information S5. The first column (Figures 6a1,a2) shows an area with no RHEED exposure (NRE) at any point throughout the growth. There are two different areas observed: a dark area and a light area. In this case, the dark area is the NaCl and the light area is GaAs. This was inferred from the continuous degradation and movement of this surface under the presence of a tightly focused electron beam in the SEM (see the details in Supporting Information S1). The difference in smoothness of the dark surface can be seen between the low and high magnification images; it was impossible to acquire a high magnification image without some roughening of the NaCl layer. However, the light areas do not degrade. The composition of these areas is not known, but they are highly ordered with the long axis nearly perpendicular to the ⟨110⟩ direction (all images in Figure 6 are oriented the same).

The images from the next three columns show areas exposed to the RHEED beam prior to GaAs deposition. Figure 6b shows an area with RE during the initial low temperature NaCl deposition but eventually covered with ∼20 nm of fresh NaCl at 300 °C while the RHEED beam was moved to the next areas. This area looks very similar to the area with NRE; it seems like 20 nm of NaCl deposition negates any effect from previous RHEED exposure. The area in Figure 6c was exposed to the beam for 180 s, which was just enough to see the RHEED pattern go slightly spotty (Supporting Information S5). Once the transition in the RHEED pattern was observed, the beam was moved to the next location, and 4.5 nm of NaCl was subsequently deposited on this location (in Figure 6c) prior to GaAs deposition. These images look mostly similar to the previous areas; there is a similar density of lighter islands contrasting against a dark NaCl background. However, the NaCl (dark) area in the low magnification image (Figure 6c1) shows two slightly different contrast areas with cracks between. This is presumed to be a roughening of the NaCl layer due to the constant exposure of the RHEED beam at 300 °C. Again, this is not seen in the area that was similarly exposed yet had ∼20 nm of new NaCl deposition (Figure 6b). Thus, it appears that 5–20 nm of subsequent NaCl deposition can hide any effects of the RHEED on the original NaCl layer. It took 180 s of constant exposure (during continuous NaCl deposition) for a change to be seen in the RHEED at 300 °C. To observe a similar change at lower temperatures takes significantly longer or is never seen. It is expected that because this significant difference is seen with 180 s of exposure, similar changes that may not be observable in SEM or RHEED are likely happening with shorter exposures and/or at lower temperatures.

The images in Figure 6d are different from all the others, supporting the hypothesis that there are effects with shorter exposures. This area was exposed to the RHEED beam for 90 s while continuously depositing NaCl (not long enough to see a change in the RHEED pattern) up until the final moment prior to GaAs deposition. Thus, this region has no fresh NaCl deposition and is the reason for the additional 4.5 nm of NaCl in the area shown in Figure 6c. Here, the morphology of both the light and dark regions are distinctly different. The lighter features are thinner than in the previous case, but still have similar directionality. The darker region shows some larger, scalelike undulations that could be related to a rougher underlying NaCl layer and no longer degrades under the presence of the SEM electron beam, similar to the next figures.

The location of the RHEED beam was moved to three different areas for the first, second, and third minute of GaAs deposition (Figure 6e–g). The dark background from each of these areas is similar and comparable to Figure 6d without the large-scale undulations. The rough island-like morphology is also observable at low and high magnifications, does not degrade under the presence of the RHEED beam at any high focus conditions, and looks similar to previous studies of stoichiometric GaAs.^30^Figure 6e shows the area with RE during the first minute; the presence of the light islands is completely suppressed. Figure 6f shows that exposure during the second minute results in light islands with a shape similar to that observed in the area with RE just prior to GaAs deposition (Figure 6d). However, the density of these islands is lower compared to areas with NRE. Figure 6g shows the area with RE during the final minute of GaAs deposition; the density of light islands is similar to regions that were never exposed at all or had fresh NaCl after exposure.

The composition and mechanism behind the formation of these light islands are still unknown and a subject of investigation. The RHEED observations (Supporting Information S5) support 3-D textured polycrystalline growth in all regions from Figure 6e–g. On the basis of the results presented, it seems that the presence of a RHEED beam, either immediately prior to or during GaAs deposition, facilitates the growth of the small dark islands in these regions (likely GaAs). The formation of the light islands seems to start immediately upon GaAs exposure, but begins to stagnate after the first minute. These could be similar to the discrete GaAs islands observed in section 2.2. The presence of RHEED facilitates nucleation of dark GaAs islands, which suppresses the formation of the light islands. Electron bombardment of NaCl surfaces was seen to promote nucleation in metal films.^21^ However, if RE is withheld until light islands have already formed (Figures 6f,g), the smaller darker GaAs islands grow around them and suppress formation of further light islands. There is no observable density of the dark GaAs islands in areas with >4.5 nm of fresh NaCl. This suggests that <5 nm of fresh NaCl is enough to erase the effects of RHEED and suppresses the nucleation of islands with this morphology at 300 °C. The effects of the RHEED beam were not limited to the growth of binary material but were also seen in the deposition of Ge on NaCl films (Supporting Information S6) where RE also enhanced the nucleation density of the Ge islands. The presence of an electron beam during material deposition results in a uniform surface morphology underneath the millimeter-wide beam. However, this would not be a practical way of achieving any large area uniformity because the nucleation of GaAs is highly sensitive to the time exposed to the RHEED beam, both in total duration and the point during the deposition.

#### 2.3.2 RHEED-Induced Arsenic Adsorption at Low Temperature

Another growth scheme was developed utilizing RHEED exposure only prior to GaAs deposition to achieve uniform nucleation in areas larger than the millimeter-wide RHEED beam. A detailed description with RHEED images is outlined in Supporting Information S7. Immediately after a NaCl layer is deposited at 150 °C, an As-flux of 12.2 atoms/nm^2^s (equivalent to the Ga flux used for the 33 nm/min growth rate) was supplied to the surface. The surface was then exposed to the RHEED beam, and the pattern went diffuse, signifying the condensation of amorphous material. The preferential condensation of amorphous As onto a bare NaCl surface was only observed in the presence of the electron beam. Thus, the beam was manually stepped across the surface until a diffuse pattern was observed over most of the sample. The total time required for this As soak (*t*_soak_) was typically 3–5 min. The substrate was then heated at a rate of 20 °C/min under constant As exposure. Around 320 °C the RHEED began to transition from diffuse to streaky as the As desorbed from the NaCl surface.

**Figure 7:**
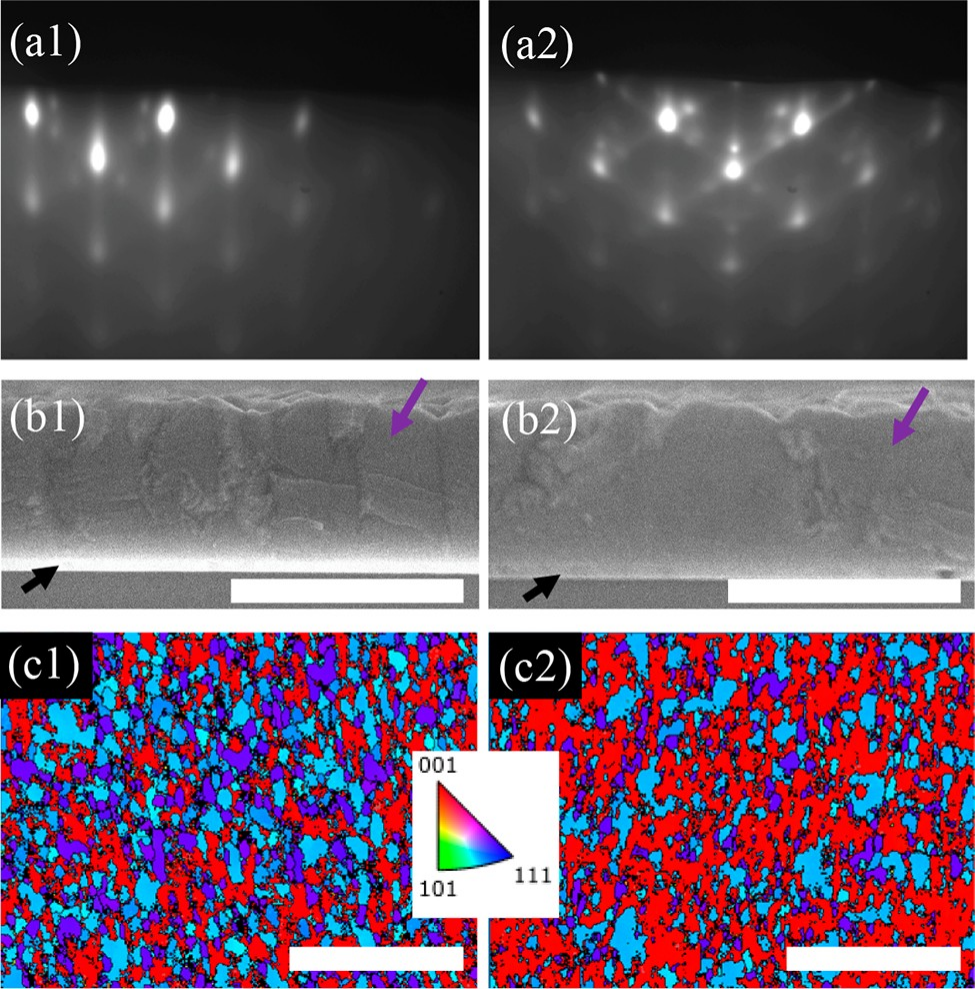
GaAs growth initialized at 350 °C and ramped to 580 °C. (a) RHEED images, (b) SEM (scale bar 600 nm), and (c) EBSD maps of areas (scale bar 5 μm) (1) exposed to RHEED and (2) not exposed to RHEED until growth was completed. Purple and black arrows mark the GaAs overlayer and NaCl layer, respectively.

Figure 7 shows the RHEED patterns, SEM, and EBSD maps of a sample utilizing this growth scheme. After ∼30 nm of NaCl deposition, the RHEED was moved across the surface in the center portion of the sample under an As flux prior to heating. Once heated to 350 °C, GaAs growth was initialized and deposited while continuously heated to 580 °C. The first column in Figure 7 shows the area with the RHEED-assisted As adsorption post-NaCl deposition. The second column shows the area with NRE. The RHEED patterns were taken at the end of growth and reveal slight chevrons in both cases. In the area with RE (Figure 7a1), the pattern is mostly spotty, with dimmer chevrons but slight streaking indicating a surface that is not quite smooth and has a lower degree of any specific faceting. The dim secondary spots suggest some sort of twin-related secondary orientation. This contrasts with the pattern from the area with NRE (Figure 7a2) which was taken only at the end of growth to avoid any influence on the structure. Here, the chevrons are much brighter and shallow angle (similar to those observed in Figure 4), with brighter shadow spots, suggesting a higher degree of twin formation. The cross-section SEM images (Figures 7b1,b2) reveal similar thickness of both the GaAs and the underlying NaCl. Perhaps the roughness in the area with NRE could correlate with the spottier RHEED pattern as well.

However, plan view EBSD (Figures 7c1,c2) reveals significantly different crystalline textures between the two areas. There are a number of different orientations observed in the area with RE, where As condensed on the surface. A portion of the grains have the same {001} out of plane orientation as the substrate (red). The shades of purple and blue are the result of different 90° azimuthal rotations of {223} grains and {122} grains, respectively. Quantitative pole figures (not shown) reveal that all {001} grains have the same azimuthal orientation as the substrate and that the prevalence of each 90° rotation of {223} is approximately equal. However, one of the rotations of the {122} grains is favored more than the other three. The EBSD data for the area with NRE shown in Figure 7c2 reveals more area oriented commensurate to the substrate (red) and significantly fewer {223} grains (purple). A quantitative pole figure of this image shows that there is an extreme preference for the same single rotation of the {122} grain (blue) that was observed in the area with RE. Because the substrates used in this study have a maximum of 0.1° offcut, the distance between atomic steps would be at minimum 160 nm. Therefore, it is possible that there is some contribution from the step edges on the grains which are only hundreds of nanometers in size. The presence of these additional grains could also explain the shadow spots observed in the RHEED patterns at the end of growth. The root cause of the grain formation, as well as the single preferential orientation of these grains, is the subject of an ongoing investigation. It is also worth mentioning that XRD is not used as a metric for crystallinity of material in this study because of the large penetration depth of X-rays. During the nucleation of GaAs on NaCl, twins and misoriented grains form (as mentioned in section 2.2.2) and result in diffraction patterns that suggest that the material is much more polycrystalline than EBSD data of the surface. Additional studies^25^ use STEM to reveal that the misorientation of these early stage grains can be reduced and eventually overgrown.

From the data shown above, one could assume that the presence of RHEED was purely detrimental for the growth of nearly single crystal GaAs on NaCl. However, as the temperature is increased the growth process with RHEED gets more complicated. Section 2.2 shows that the NaCl film is highly volatile and can completely desorb if not sufficiently capped before heating to elevated temperatures. A persistent NaCl layer must be maintained if one hopes to achieve liftoff of the overlayer. Section 2.3.1 shows that RHEED promotes the faster nucleation of GaAs, which enables more rapid coalescence to protect the NaCl at higher temperatures.

#### 2.3.3 Separate Low-Temperature Nucleation and High-Temperature Growth

The final growth scheme for this investigation was devised using everything shown until this point in an effort to achieve more crystalline GaAs overlayers (Supporting Information S2c). After NaCl deposition at 150 °C, the sample was exposed to an As-flux and the RHEED beam manually stepped across half of the sample surface. It was then heated to 400 °C, and ∼100 nm of GaAs was deposited at a rate of 33 nm/min. However, now the growth is paused, and the sample is heated to 580 °C. Once the sample reaches this temperature, 300 nm of GaAs is deposited.

**Figure 8:**
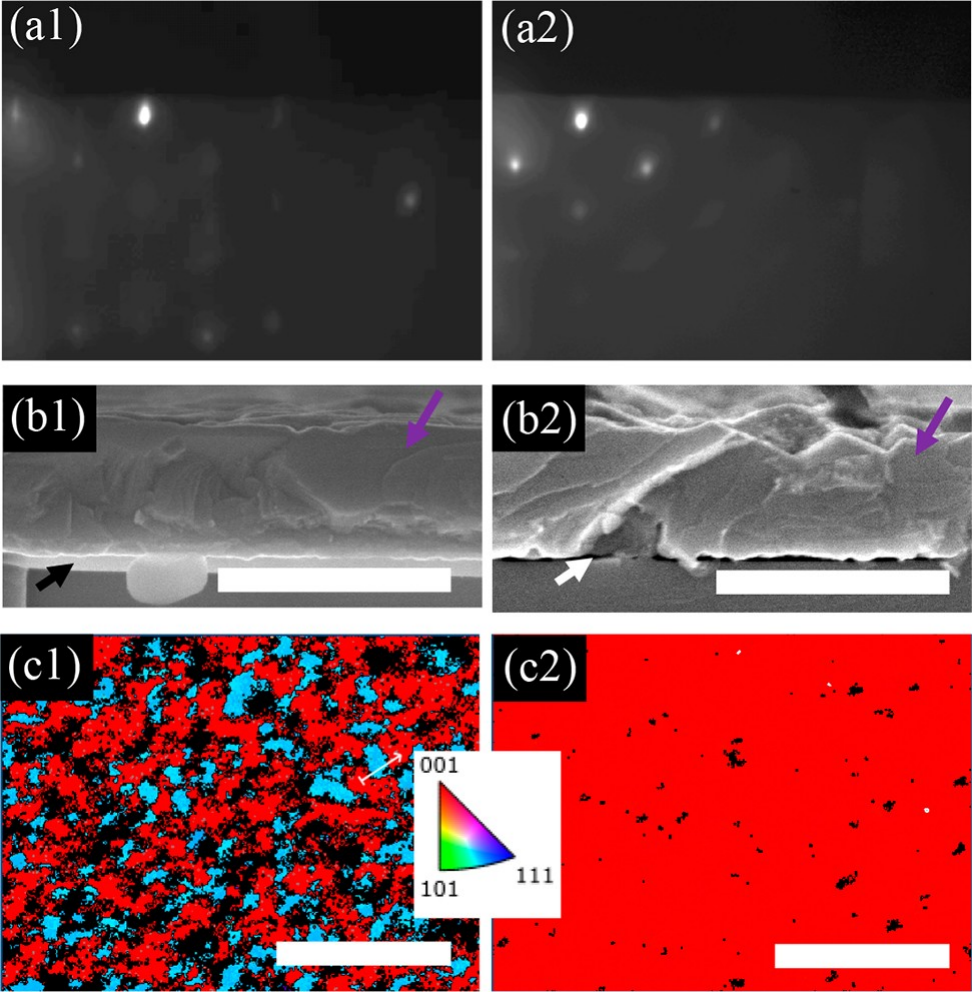
(a) RHEED images at the end of growth, (b) SEM images where purple, black, and white arrows mark the GaAs overlayer, NaCl layer, and voids between the substrate and overlayer, respectively (scale bars 600 nm), and (c) EBSD maps of areas (scale bars 5 μm) (1) exposed to RHEED and (2) not exposed to RHEED until growth was completed of a sample with a 3 min GaAs nucleation at 400 °C with additional 9 min of growth at 580 °C.

The RHEED patterns from areas with RE and NRE are shown in Figures 8a1,a2. The pattern from the area with RE is slightly streakier, while the pattern from the area with NRE shows faint steeper chevrons. This suggests that the area with NRE has a rougher faceted surface compared to the area with RE. Both areas are a bit dimmer but have substantially fewer additional spots than the samples in section 2.3.2 that were grown continuously from the nucleation at lower temperatures. Parts b1 and b2 of Figure 8 display SEM images that show a stark contrast between the two regions. A complete NaCl layer of the expected thickness is still present in the areas with RE. Conversely, in the area with NRE, there is no more NaCl and the overlayer has fused to the substrate. The area with NRE is unsurprisingly similar to samples grown at similar temperatures discussed in section 2.2.1 (such as Figure 3, which showed fusion with the substrate when nucleating as cold as 325 °C), while the area with RE is quite different. By utilizing the RHEED exposure at low temperature, GaAs was nucleated more quickly at 400 °C, resulting in more rapid coverage of the NaCl, which was protected even during the high temperature deposition. Additionally, a relatively uniform area larger than just the width of the RHEED beam was able to be achieved because this RHEED sweep was done prior to any GaAs deposition (see the image of the sample in Supporting Information S8a).

The EBSD maps in Figure 8c1 show that there are only two predominant grain orientations in the RE area, those oriented commensurate to the substrate and a single orientation of the {122} (quantitative pole figure in Supporting Information S8b). The {223} grains that were previously observed are now fully suppressed. The origin of this orientation must be a result of the lower GaAs nucleation temperature or the continuous deposition of GaAs while heating. The EBSD from the area with NRE (Figure 8c2) shows monocrystalline GaAs. Because the material in this region does not show a complete NaCl layer, this could be a result from the bonding to the substrate, either homoepitaxy, recrystallization, or a combination of both. Without the presence of a NaCl layer it will not be possible to be simply removed from the substrate anyway. The increase in the amount of (100) overlayer material in the RE case is encouraging. By carefully tuning additional growth parameters, we have realized large-area monocrystalline GaAs films on top of complete NaCl layers in a separate report.^25^ In this case, the continuous NaCl layers enable the removal of the GaAs overlayer.

#### 2.3.4 Discussion of RHEED Effects

The RHEED effects of only three samples of GaAs on NaCl are shown in the previous section, but a large number of samples (>200) have been systematically grown and analyzed including some that deposit Ge directly on NaCl surfaces (Supporting Information S6). They reveal that the effects of RHEED are quite complex and neither exclusively beneficial nor detrimental. Some general observations and conclusions are outlined below:

##### The Presence of RHEED Roughens NaCl

This effect is likely reduced with reducing the accelerating voltage, but then the pattern is too dim to be interpreted. This effect also seems to be more pronounced at higher temperatures; i.e., at 15 kV it takes ∼180 s for the RHEED pattern to transition from streaky to spotty at 300 °C, but at 150 °C even after exposure for significantly longer times the RHEED is unchanged. Previous reports have suggested that the electron beam results in dissociation and desorption of the NaCl.^21,31^ This could also be why imaging bare NaCl layers under highly focused electron beams in SEM and TEM results in the changes and destruction of the NaCl discussed in Supporting Information S1. Unfortunately, most other *ex situ* techniques to look at more subtle changes between areas with RE cannot be used on bare NaCl films because the NaCl degrades appreciably in the presence of water vapor, even in the amount present in the air.

##### RHEED and Delamination

The presence of the RHEED sometimes causes delamination. This is most frequently observed at the edges of the RHEED exposed areas. For samples with deposition temperatures >350 °C, the area with RE shows a complete NaCl layer beneath a GaAs overlayer, while the area with NRE shows frequent fusion to the substrate. Delamination is somewhat obviously not observed in areas that have frequent fusion to the substrate, but it is also never observed in the regions that have a complete NaCl layer. However, at the transition between these two regions, the film is occasionally observed to be pulling away from the substrate; there is no NaCl and the fusion to the substrate is less frequent. For samples which use the As-sweep technique this is thought to occur because this transition region only somewhat protects the NaCl due to less enhanced GaAs nucleation compared to areas with longer, more deliberate RE. Thus, this area has larger islands, which the NaCl sublimates out from underneath during prolonged high temperature steps, resulting in larger voids. This is especially true for films with thin nucleation layers heated to high temperatures for thicker (>300 nm) high temperature grown layers. This effect is also observed in the case with prolonged RHEED exposure. In this case it could be the result of the RHEED roughening the film, discussed in the previous point, to the extent that holes are formed completely though the NaCl layer, which allows for less frequent fusion of the overlayer to the substrate and subsequently delaminates.

##### Excessively Long RHEED Exposure Times Do Not Enhance Crystallinity of GaAs

While some beneficial effects may be gleaned from selective exposure, prolonged exposure never seemed to give desirable results. This could be due to surface roughening or related to the higher degree of polycrystallinity observed in areas with RE. Constant exposure during the GaAs deposition resulted in a more polycrystalline material and sometimes spontaneous delamination of the film during growth.

##### The Presence of RHEED Enhances Arsenic Adsorption

As discussed in section 2.3.2, arsenic preferentially condenses where the RHEED beam is present. The reason is not fully understood, but it is possible this is from a slightly rougher surface facilitating nucleation points, or from a change in surface charge promoting adhesion of As adatoms. As the temperature is increased, this amorphous As layer has to desorb before the NaCl surface atoms can, so in some way it can protect the NaCl at elevated temperatures. However, they both begin to desorb at similar temperatures, so the impact is limited. Related to the previous points, if the bare NaCl is exposed to the RHEED beam for too long the overlayer tends to delaminate from the substrate.

##### The Presence of RHEED Promotes Nucleation of GaAs on NaCl

This seems to be true not just during the actual nucleation step, but even exposure prior to opening the Ga shutter with the As adsorption. It is not known whether this is due to slight roughening of the NaCl or from the actual As-adsorption itself as it may not necessarily be fully desorbed by the time the Ga shutter is opened. However, this enhanced nucleation rate is one of the key benefits of RHEED for the formation of higher quality GaAs films on NaCl because swift formation of a complete GaAs layer is crucial to enable higher temperature depositions without sublimation of the NaCl layer.

##### RHEED Affects the Crystallinity of GaAs Grown on NaCl Using Traditional Codeposition Techniques

In the cases discussed above it is possible that RHEED (or at least the As adsorption) is a cause of the highly textured GaAs overlayer because RE areas have more of the {223} grains than areas that are not exposed. For some samples such as the one where GaAs was deposited continuously from 250 to 580 °C (Figure 3c), EBSD revealed that areas with RE showed grain sizes >5 μm, while the grains in areas with NRE were only a few hundred nanometers. However, there were no grains oriented commensurate to the underlying substrate or NaCl layer in either area. This could be related to previous studies on electron beam annealing of amorphous GaAs,^32,33^ but these studies were done with much higher accelerating voltages.

## 3 Conclusion

In conclusion, thin NaCl layers have been deposited on GaAs (001) substrates, as well as GaAs on top of the NaCl. The temperature at which the GaAs is nucleated has great effects on the crystallinity and overall morphology of the end film ranging from discrete islands, to extremely porous interfaces, to fully dense films with sharp interfaces. The presence of a RHEED beam, both prior to and during GaAs nucleation, can profoundly change the structure of the overlayer. This is in large part because of the enhanced nucleation of GaAs islands, enabling more rapid coalescence of a GaAs film to protect the volatile NaCl layer. Through combining careful RHEED exposure and separating low-temperature nucleation and high-temperature growth of the overlaying GaAs layer, highly textured GaAs films on persistent NaCl layers were achieved.

## 4 Methods

An Epi930 MBE chamber is used to deposit NaCl layers on GaAs (001) ± 0.1° substrates. The GaAs substrate is heated to 620 °C for 25 min under exposure to As before growth to ensure a clean and oxide-free surface prior to deposition of a 300 nm GaAs buffer layer at 580 °C. Ga is supplied from a standard effusion cell and As is provided by a valved cracker source. The substrate is then cooled under an As overpressure until ∼340 °C, after which the As is closed, and it is cooled to the temperature of NaCl deposition. NaCl is deposited via sublimation of 5 N NaCl from a conventional effusion cell. Deposition temperatures investigated for the growth of a NaCl layer and the subsequent GaAs layer range from 100 to 350 °C and 100–580 °C, respectively. The temperature is measured via band-edge thermometry using a kSA BandiT system. RHEED with an accelerating voltage of 15 kV is used to measure the surfaces during growth.

The growth morphology and epitaxial relationships of the different layers were investigated using various methods of electron microscopy. SEM was performed on a Hitachi S-4800 using accelerating voltages from 3 to 7 kV and a beam current of 5–7 nA. EBSD data was acquired with the sample tilted at 70° using an Oxford system with a Symmetry detector and CMOS sensor technology and an acquisition voltage of 20 kV. TEM imaging, electron diffraction patterns, and EDX were carried out with a JEOL 2100F/Oxford Instruments X-Max EDX at 200 kV. The GaAs substrate was tilted so that incident electrons are along ⟨110⟩. Bright field TEM imaging was performed to show both overall layers and atomic structure of defects. Electron diffraction within an area of 100 nm in diameter was acquired to identify the local phases and crystalline orientation. To minimize damage from long electron beam dwell times during EDX map acquisitions, the average information across regions of interest was collected. EDX mapping was conducted with Aztec in ≤10 min. Subsequently, the counts of the EDX map were summed along the horizontal line perpendicular to the growth direction to achieve line profiles of elemental distribution.

## Supporting Information Available

Discussion of the fragility of NaCl layers in air, in the presence of water, and under electron beams in the SEM; schematics of sample growths including shutter sequencing and temperatures; behavior of NaCl layers during high temperature ramp/anneal; exclusively low temperature growth of GaAs on NaCl (without anneal); details on multi-RHEED exposure sample; deposition of Ge on NaCl thin films and RHEED effects; discussion of low temperature As adsorption and RHEED images; quantitative pole figures(PDF)
