# Supplementary material for: Consideration of the Intricacies Inherent in Molecular Beam Epitaxy of the NaCl/GaAs System
Source: ACS Omega. 2022 Jul 1;7(28):24353–64. doi: 10.1021/acsomega.2c00954 (PMC9301643; doi:10.1021/acsomega.2c00954)
Supplement: Supplementary file 1 — ao2c00954_si_001.pdf [file ao2c00954_si_001.pdf]

Consideration of the Intricacies Inherent in Molecular Beam Epitaxy of the NaCl/GaAs System

Brelon J. May[1], Jae Jin Kim[2], Patrick Walker[1], William E. McMahon[1], Helio R. Moutinho[1], Aaron J. Ptak[1], David L. Young[1*]
[1]National Renewable Energy Laboratory, Golden, USA
[2]Shell International Exploration and Production Inc., Houston, USA

SUPPORTING INFORMATION

S1. Discussion of the fragility of NaCl layers in air, in the presence of water, and under electron beams in the SEM

S2. Schematics of sample growths including shutter sequencing and temperatures

S3. Behavior of NaCl layers during high temperature ramp/anneal

S4. Exclusively low temperature growth of GaAs on NaCl (without anneal)

S5. Details on multi-RHEED exposure sample

S6. Deposition of Ge on NaCl thin films and RHEED effects

S7. Discussion of low temperature As-adsorption and RHEED images

S8. Quantitative pole figures

S1. Fragility of NaCl layers in air, in the presence of water, and under electron beams in the SEM

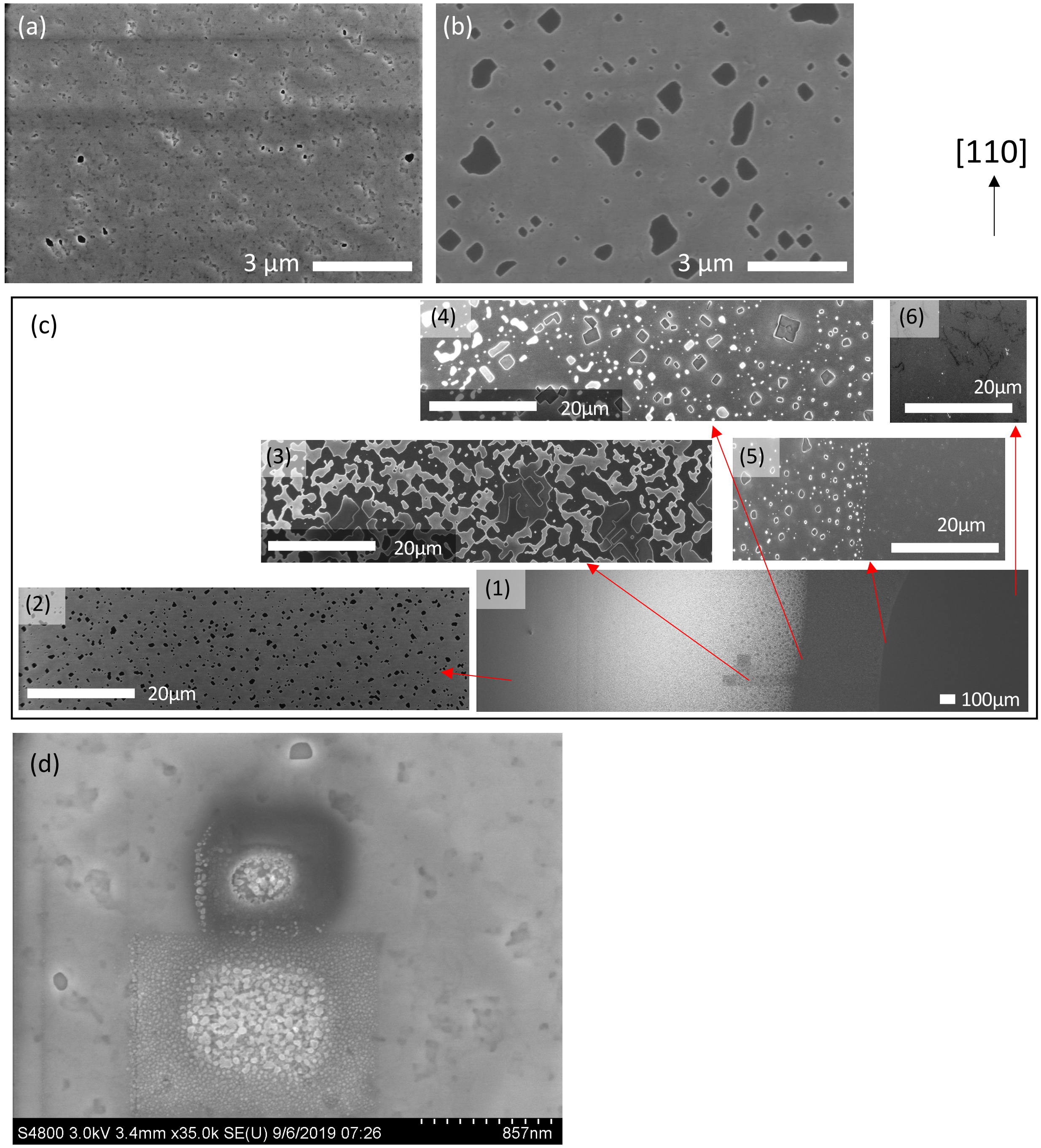

Figure S1: Plan view SEM images of a bare NaCl thin film deposited on GaAs. (a) Image of the sample the day of growth and (b) 4 days after growth with most of the time in a nitrogen dry box. (c) Images of the sample the day of growth that was partially dipped in water for ~1s. (1) The low magnification image shows three distinct regions with the right side being the side dipped into water. Higher magnification images of the different regions (2-6) show lighter colored NaCl and the darker GaAs substrate. (d) Images of a NaCl surface with a 3kV beam at low magnification showing regions that were roughened form exposure at higher magnification

The main manuscript discusses that the *in*-situ RHEED beam used for characterization has effects on the NaCl layer. However, bare NaCl layers are also extremely difficult to measure using *ex-situ* diagnostic tools. The {100} facets may be the low energy facet, and mostly stable in air, as one can see when using normal table salt. However, there are effects at small length scales that happen rapidly when removed from vacuum which has proved difficult for doing detailed analysis of bare NaCl layers. Figures S1 shows a variety of plan view SEM images of a 90 nm bare NaCl film deposited on a GaAs substrate. Figure S1(a) shows an image of the sample immediately after growth, and exposed to air while moving to the SEM. The surface is not as smooth as RHEED images would suggest. Some holes are also observed, it is not known whether these holes go all the way to the GaAs substrate, whether they form during growth or the walk over to the SEM. Figure S1(b) shows the same sample after storage in a nitrogen drybox for 4 days. The density and size of holes is now much larger. The sample is mounted with a cleaved {110} edge parallel to the sample holder, and these holes show very specific edges, which at ~45°, are likely the low energy {100} facets of NaCl. How these form in a dry environment is not a subject of this study, but rather here we focus on pointing out the difficulties in achieving consistent measurements between samples.

It is known that NaCl dissolves readily in water, but the extent to which thin films can be removed, and at what speed was tested here. The dissolution of NaCl was also investigated by partially submerging a piece of the bare NaCl (after storage in the drybox for 4 days). The sample was dipped in room temperature water by hand and immediately removed and blown dry with nitrogen before doing additional plan view SEM (Figure S1(c)). In the lowest magnification image (Figure S1(c1)) the right side of the image was dipped in water and the left was not; there are three clearly different contrast regions. Furthest from the water, in area (2), the NaCl surface still shows a large density of square holes resembling those seen in Figure S1(b). Moving further right, closer to the dipped area, the prevalence of the holes increases, and eventually the sample is only ~50% covered with salt (area (3)). Here, the NaCl still shows preferential directions with right angle faceting. Moving even closer to the edge (area (4)), one is left with discrete NaCl islands. These islands still have similar 90° edges and is likely right on the edge of where the water reached. Moving further right into the middle area, the islands no longer have such strong faceting or relationship to the substrate as seen in the left side of Area 5. This could be due to either the significantly higher vapor pressure of water at <1 mm from the surface in a dry environment (Denver, CO), instability during the water dip, or in the meniscus of the water surface, which could dissolve the NaCl, but then recrystallize as the highly NaCl-rich solution that was purely at the surface is dried. Beyond this line, nothing appearing like NaCl is observed suggesting that it is fully dissolved. There is some contrast/scratching on the GaAs surface but it is believed that a GaAs surface similar to the as-received case could be achieved by more carefully rinsing the surface with water, and subsequent thermal cleaning in vacuum and buffer layer growth. This all suggests that NaCl could provide a suitable release layer, but also study of bare layers in high humidity environments would be extremely difficult.

The final effect discussed in this section pertains to Figure S1(d) and is the fragility of the NaCl under exposure to electron beams. Here, another plan view SEM image is shown, taken with an accelerating voltage of 3 kV and beam current of 6.2 nA. Prior to acquisition of this low magnification image, the beam was focused in the two regions at a higher magnification (top) and medium magnification (bottom) which show significant effects from the electron beam. One can watch the surface move and evolve in real time while focused on these areas at higher magnification. Adjusting the focus and stigmation of the incoming beam i.e. oscillating the depth

of focus, results in faster decay of the surface and deep holes can be made. It is not known whether this material is ablated, moved aside, or is different in crystallinity or composition than the original material. Additionally, as with the medium magnification area, using a long exposure time can result in degradation of the entire area. Thus, in order to acquire an image of a lesser damaged surface, one must move from the focused area to a new area and acquire the next image quickly. These effects are seen at accelerating voltages 3-5× lower than what is used in RHEED. It is possible that even though the electron beam in RHEED is at a glancing angle, rather than directly impinging on the NaCl surface, the degradation observed (such as observed in Figures S5(c,d)) could be because of a similar mechanism. The roughening of the surface observed via SEM could be comparable to what is happening when the surface is exposed to RHEED during the growth as well.

S2. Schematics of sample growths including shutter sequencing and temperatures

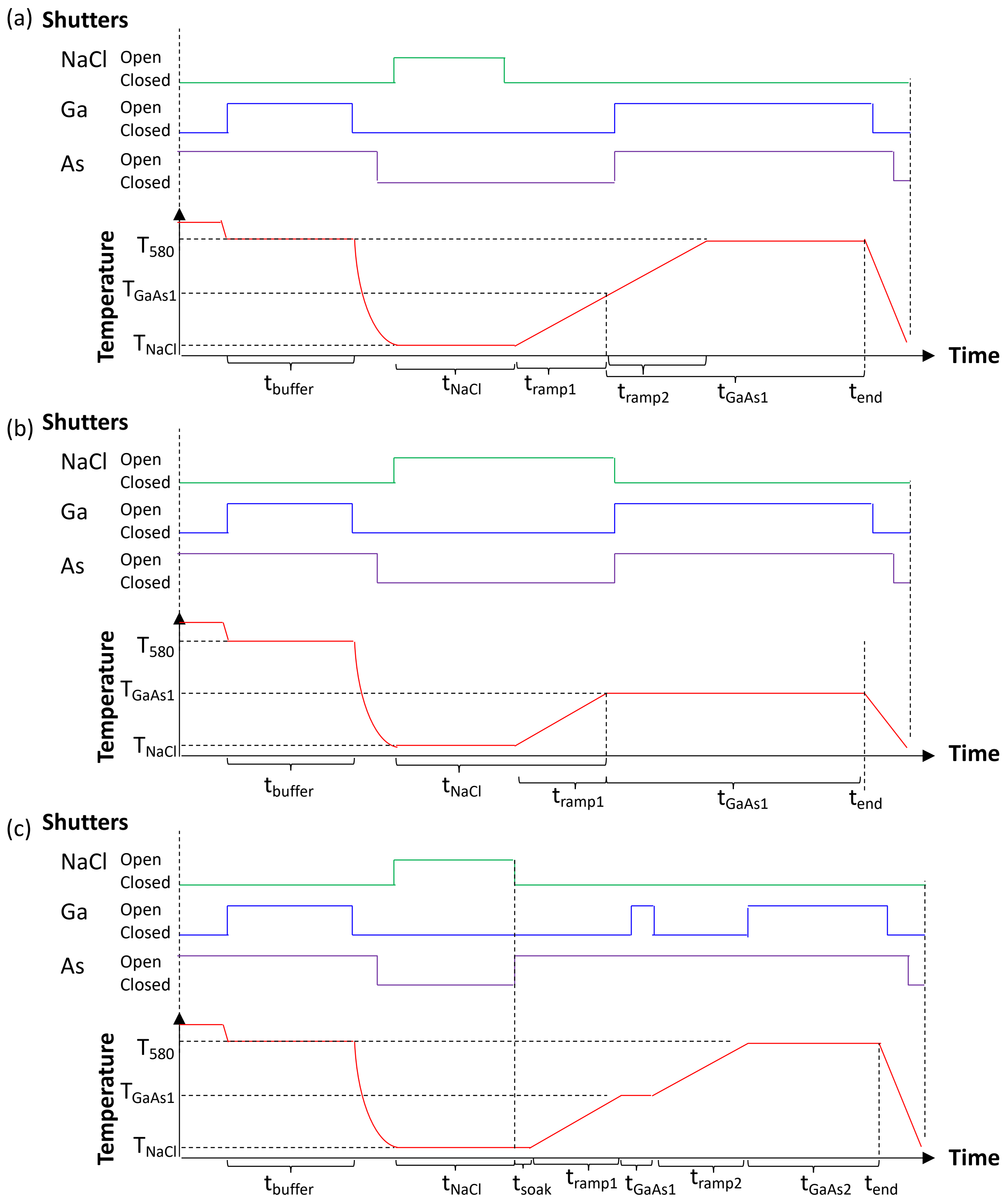

Figure S2: Growth schematics showing shutter positions for NaCl (green), Ga (blue), and As (purple), with growth temperature (red) as a function of time for the growths discussed in (a) Figure 3, (b) Figure 4, and (c) Figure 8 in the main text.

Variable	Definition
T_{580}	580°C
T_{NaCl}	Temperature of starting NaCl deposition
T_{GaAs1}	Temperature at which the first GaAs deposition begins
t_{buffer}	time of buffer layer GaAs deposition (9 minutes)
t_{NaCl}	time of NaCl deposition
t_{ramp1}	time taken to increase temperature from initial NaCl deposition temperature (T_{NaCl}) to GaAs nucleation temperature (T_{GaAs1})
t_{ramp2}	time taken to increase temperature from the GaAs nucleation temperature (T_{GaAs1}) to 580°C
t_{GaAs1}	total time of first initial GaAs deposition
t_{GaAs2}	total time of second GaAs deposition
t_{soak}	time taken to sweep RHEED across NaCl surface under As-flux
t_{end}	end of growth

Table S1: Definition of variables outlined in Figure S2

Figure S2 shows schematics of the processes for the GaAs-capped growths discussed in the main manuscript. The shutter sequences for NaCl (green), Ga (blue), and As (purple) are shown in conjunction with the temperature of the substrate (red), as a function of time beginning from the oxide desorb step at 620°C, where the As shutter is already open. All samples go through this identical step, as well as the same time for a buffer layer (t_{buffer}) of 9 minutes to achieve a thickness of ~300 nm at 580°C (T_{580}), prior to cooling rapidly under an As flux until ~340°C. The samples are all then cooled until the temperature at which NaCl deposition begins (T_{NaCl}). The salt deposition time (t_{NaCl}) is varied between studies, and sometimes persists into the time taken to ramp temperature (t_{ramp1}) to the GaAs nucleation temperature (T_{GaAs1}). The substrate temperature ramp rate post-NaCl deposition is either 20 or 50°C/min depending on the sample set.

Figure S2(a) represents the growth scheme for Figure 3 in the main text. The NaCl is deposited only at low temperature. For these growths the ramp rate after NaCl deposition is 50°C/min. Thus, the time to ramp from T_{NaCl} to T_{GaAs1} (t_{ramp1}) and from T_{GaAs1} to T_{580} (t_{ramp2}) are both dependent on the T_{GaAs1} chosen; $t_{ramp1}+t_{ramp2}\approx(580°C-T_{NaCl})/(\text{ramp rate})$. However, this is only approximately equal because it can take up to 5 minutes before the heater actually increases the substrate temperature. Additionally, the GaAs deposition time (t_{GaAs1}) is also dependent on T_{GaAs1} as it includes t_{ramp2} and the time of growth at 580°C. However, the time at 580°C is lengthened/shortened in an effort to keep the total thickness of the layers the same between sample comparisons, i.e. t_{GaAs} is attempted to be kept the same for all samples. For these samples, once T_{GaAs1} is reached, both the As and Ga shutters are opened simultaneously. The Ga shutter remains open for t_{GaAs1}, before closing and cooling the sample at the end of growth (t_{end}). The sample cools under an As-overpressure until ~340°C when the As shutter is also closed.

Figure S2(b) is a somewhat simpler scheme pertaining to Figure 4. The first part of the growth is the same as in Figure S2(a). However, in this case t_{NaCl} also includes t_{ramp1} as the NaCl shutter is kept open while ramping the substrate temperature, and a ramp rate of 20°C/min is used.

Thus, the thickness of the NaCl deposited is necessarily dependent on both t_{NaCl} and the T_{GaAs1} chosen. The temperature stops increasing once T_{GaAs1} is reached. At this point the NaCl shutter is closed, and both Ga and As are immediately opened. The Ga shutter remains open for t_{GaAs1}. Once the growth is completed, the Ga shutter is closed. If T_{GaAs1}>340°C, the sample is cooled to ~340°C prior to closing the As shutter. If T_{GaAs1}<340°C, the Ga and As shutters are closed simultaneously.

Figure S2(c) pertains to Figure 8 in the main text and includes a two-step GaAs deposition and a time where arsenic is exposed to the NaCl surface. The time it takes to move the RHEED beam manually across the surface to effectively dim the diffraction pattern is t_{soak}. During this time, the As shutter is opened while the sample is still at T_{NaCl}. The As shutter remains open using an As flux with a As/Ga ratio = 1 (calibrated at 580°C) while heating the sample to T_{GaAs1} at 20°C/min (which takes a predictable amount of time (t_{ramp1}). Once the T_{GaAs1} is reached, the temperature stabilizes, and then the Ga shutter is opened for a short time (t_{GaAs1}) to form a thin layer at this lower temperature. The As shutter remains open as it is heated to T_{580} using the same ramp rate (which takes t_{ramp2}) at which point the As flux is increased and the Ga shutter is opened for a secondary GaAs deposition time (t_{GaAs2}). Once complete, the same cooling procedure as outlined earlier is then applied.

This scheme can also be applied to Figure 7 in the main text (and Supplementary Figure S7). In this case there would be no growth pause, so t_{GaAs1}=0, and the Ga shutter would be open for the entirety of t_{ramp2}.

S3. Behavior of NaCl layers during high temperature ramp/anneal

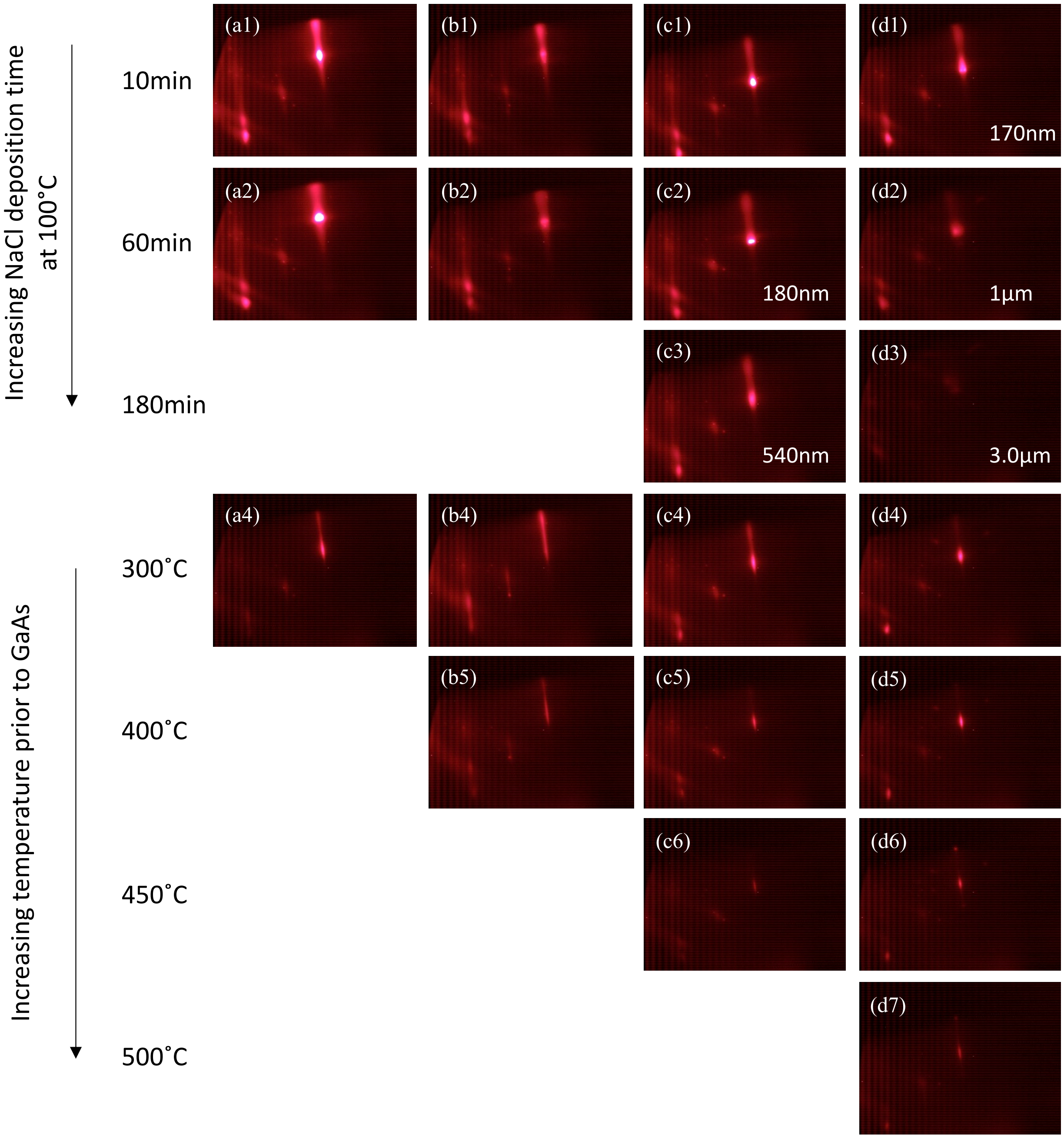

Figure S3: Additional RHEED patterns along the <110> for samples discussed in Figure 4 in the main text consisting of NaCl deposited for various times at 100°C and capped with GaAs at various temperatures. (a) 72 min of salt capped at 350°C, (b) 74.5 min of salt capped at 400°C, (c) 197 min of salt capped at 450°C, (d) 199.5 min of salt capped at 500°C. RHEED images taken at (1) 10 min, (2) 60 min, and (3) 180 min into NaCl deposition at 110°C (where applicable) and of the NaCl surface with continuous NaCl deposition upon heating to (4) 300°C, (5) 400°C, (6) 450°C, and (7) 500°C.

This section shows RHEED patterns taken prior to those shown in Figure 4 in the main text. As discussed in Supplementary section 2, for this growth scheme the thickness of the NaCl changes as it is based on t_{ramp1}. Additionally, thicker NaCl was required to maintain a layer at high

temperatures. For growth temperatures (T_{GaAs1}) up to 450˚C (Figures S4(a-c)), this was achieved by simply growing thicker NaCl layers using a growth rate of ~3 nm/min at 110˚C (corresponding to a beam equivalent pressure (BEP) of ~7.3e-8 torr) and increasing the deposition time of NaCl (t_{NaCl}). To achieve a persistent NaCl layer with growth of GaAs at 500°C in a reasonable amount of time the growth rate of the NaCl was increased, the BEP of NaCl was increased to 4e-7 torr and NaCl was deposited for 180 min at 110°C (plus a t_{ramp1} ~19.5 minutes for increasing the temperature from 110-500°C). Neglecting any desorption, this would be equivalent to growth of a layer that is ~3.3 μm thick.

Looking at the RHEED patterns for the lower growth rate samples, the patterns after 10 min (Figure S4(a1-c1) are the same as after 60 min of deposition (Figures S4(a2-c2)). For the case of higher NaCl growth rate (Figures S4(d1-d2)) the pattern dims slightly, although at this point there is already >1 μm of material, and even at a lower growth rate, after 540 nm of material (Figure S4(c3)) the pattern started to dim. So it is possible that this dimming is only related to total thickness of the NaCl and not the higher growth rate.

For the lower growth rate samples, heating to 300°C and beyond shows a dimming of the RHEED pattern, which continues until T_{GaAs1} is reached, and the GaAs capping begins. In the case of higher deposition rate required for T_{GaAs1}=500°C the RHEED pattern is slightly dimmer during the growth. However, the pattern begins to brighten as the temperature is increased to 300°C (Figure S4(d4)). This could possibly be attributed to a slight annealing of the layer at temperatures <300°C before the NaCl layer starts to desorb. But similar to the previous cases, the RHEED pattern begins to dim at >300°C, likely due to the increasing desorption and decomposition of the NaCl. In all cases there is at least a weak NaCl pattern still observable prior to beginning GaAs nucleation.

S4. Exclusively low temperature growth of GaAs on NaCl

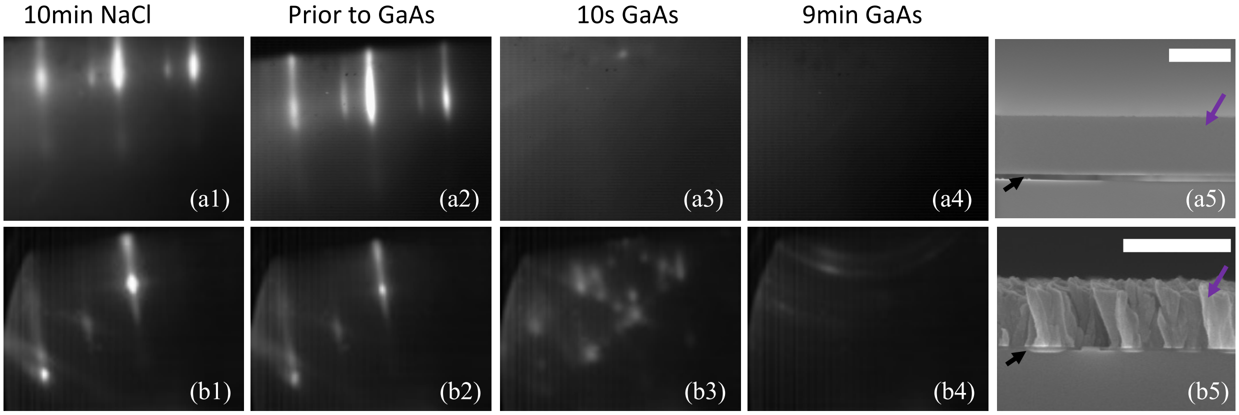

Figure S4: (Top) RHEED images taken along a <100> direction (a1) after 10 min and (a2) 30 min of NaCl deposition at 110°C, and (a3) after 10 s and (a4) 9 min, of GaAs deposition at 110°C with (a5) a corresponding cross sectional SEM image of the growth.
(Bottom) RHEED images taken along a <110> direction (b1) after 10 min of NaCl deposition at 100°C and (b2) the NaCl surface once heated to 250°C just prior to GaAs deposition. Additional patterns (b3) after 10 s, and (b4) after 9 min of GaAs deposition with (b5) a corresponding cross sectional SEM image of the growth. Scale bars in both SEM images are 600 nm.
Purple and black arrows show the GaAs overlayer and NaCl layer, respectively.

Some samples similar to those shown in Figures 3 and 4 in the main text were grown to look into the structure of purely low-temperature deposited GaAs on NaCl. These sample have no heating during the GaAs deposition, similar to Figure 4, but do not have continuous NaCl deposition. The RHEED again suggests that the NaCl layer is smooth, near single crystalline, and aligned with the substrate in both cases.

For the case of all 110°C deposition, the secondary spots in the RHEED patterns to the left of the peaks in the top row are not secondary phases or reconstructions and are due to the incoming beam having two primary spots two during this growth. The pattern from the NaCl surface quickly disappears upon opening of the Ga and As shutters and the pattern remains diffuse throughout the entirety of the growth. However, unlike the sample shown in Figure 3(a) in the main text, because this sample was never heated, a dense film with sharp interfaces is observed (Figure S3(a5)). TEM and EDS measurements (not shown) reveal that this deposited material is completely amorphous and As-rich.

The RHEED patterns in Figure S3(b) remain relatively unchanged when heating the NaCl to 250°C. However, without the continuous heating, the RHEED transitions from the streaky NaCl surface to showing many spots and chevrons, similar to patterns observed with higher temperature depositions in Figure 4 from the main text. First, the transition to a spotted RHEED indicates the formation of a three-dimensional surface. The chevrons passing through the first order spots viewed along [110] suggest GaAs islands take on a pyramidal shape with {111} facets. Extra spots symmetric about the primary and first order reflections also appear very early on in the GaAs deposition indicating the presence of twinning along the <111> directions. As the growth continues, this pattern fades and is replaced with rings with diffuse spots (Figure S3(b4)) similar to that observed during crystallographically textured nanowire formation.[26] SEM of this sample (Figure S3(b5)) shows complete coverage of a NaCl film with material consisting of densely

packed but discrete columnar grains. This is in stark contrast to Figure 3(c) in the main text where growth is initialized at the same temperature, but continuously heated to 580°C.

S5. Details on multi-RHEED exposure sample

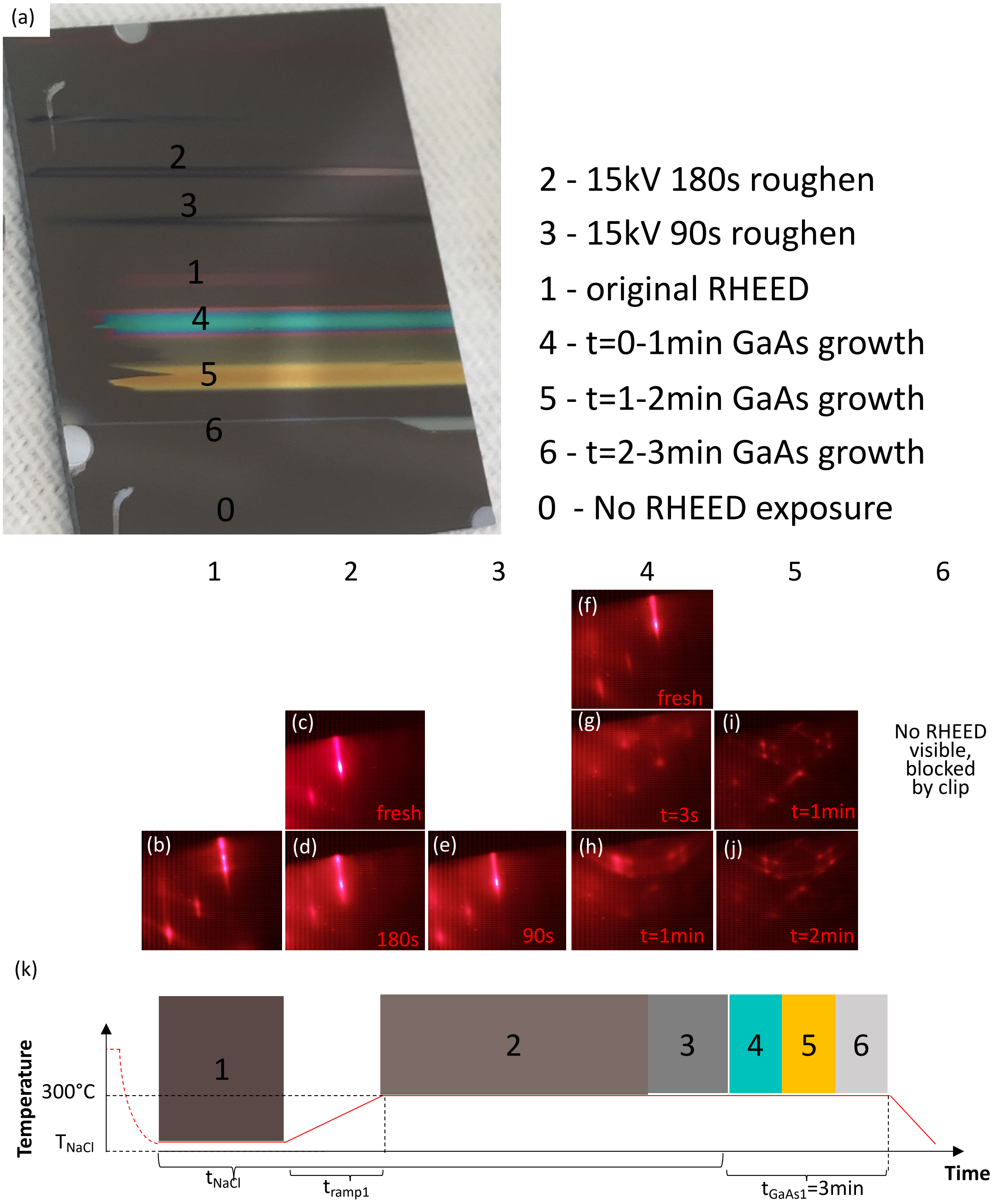

Figure S5: (a) Image of a sample after removal of the chamber showing distinct lines from exposure to the RHEED beam during different portions of the growth (labeled 0-6). (b-j) RHEED patterns from the different stages of growth in regions 0-6. (k) A representation of timing of the RHEED exposure for areas 1-6 from (a).

Figure S5 contains details of the sample from Figure 6 in the main text, consisting of a 3 min GaAs deposition on NaCl at 300°C. A picture of the sample (Figure S5(a)) shows distinct color differences from the presence of the 15 kV RHEED beam at different portions of the growth. In the picture of the sample, areas exposed prior to GaAs deposition (lines 1 and areas above) appear relatively similar, while the three regions at different points during the GaAs growth (4, 5, and 6) have drastically different colors. These also presented morphological differences in plan-view SEM, which enabled acquisition of images with a high degree of spatial accuracy for Figure 6.

Area 0 has no RHEED pattern because it was never exposed, and Area 6 was blocked by the clip so no pattern could be obtained, so these will not be discussed. However, for Area 6 there was a region near the edge of the sample where the clip no longer shadowed the beam that was used for the plan-view SEM discussed in the main text. Figure S5(b) shows the pattern after 10 min of NaCl deposition at 110°C, it appears streaky as was observed in other growths. At this point the RHEED was moved to the edge of the sample (top of sample in Figure S5(a)) and turned off while the sample was heated (with continuous NaCl exposure). Figure S5(c) shows the brightened pattern of a fresh area of NaCl (during deposition) once the temperature reached 300°C. The beam is left on and unmoved while constantly monitoring for any change. Under continuous NaCl growth, it takes ~3 minutes for the RHEED pattern to begin to go spotty as shown in Figure S5(d) signifying that the surface was roughened. At this point the beam was moved to position 3 where it is left on for 90s. The pattern after holding the beam at Area 3 for 90s is Figure S5(e), and unsurprisingly looks like it would fall between Figure S5(c) and S5(d).

GaAs deposition began immediately once the beam was moved to the fresh NaCl surface in Area 4 (Figure S5(f)), which at first looks bright and streaky. The pattern after 3s of GaAs deposition (Figure S5(g)) shows the formation of new dim spots, with faint chevrons and shadow spots. After 1 minute (Figure S5(h)) the pattern has transitioned to spotty rings. This signifies that the growth starts out oriented but three dimensional, and after 1 minute of growth with continuous RHEED exposure ends up as a textured polycrystalline film. The beam is then moved to area 5 (Figure S5(i)) which does not show any ring-like characteristics. Rather, a spotty pattern similar to the onset of GaAs deposition with RHEED exposure (Figure S5(g)) is observed. However, the pattern is much less blurred, and the spots and chevrons are seen clearly. After a minute of continues deposition with exposure, rings start to appear again. This signifies that upon further deposition with RHEED exposure the growth starts to go more polycrystalline. Without the presence of the RHEED beam during nucleation, the clearer pattern observed suggests that the RHEED may be worsening the crystallinity of the nucleation layer. However, even without constant exposure to the RHEED beam, further deposition at 300°C would be polycrystalline, similar to Figure 4(a2) in the main text which was a longer growth performed at 350°C.

Figure S5(k) outlines the portion interest for the growth of this sample, neglecting oxide desorb and buffer layer growth steps, while highlighting the timing of the RHEED exposure in relation to the NaCl and GaAs deposition. The time axis is roughly to scale. The RHEED was not open for the entity of the time marked by region 1, and was turned on and off throughout this portion. However, for the remaining sections the RHEED was left on without any beam blanking. Note that NaCl is deposited for a considerable time (~270 s) at 300°C, t_{NaCl} spans beyond the temperature increase, contrary to previous growths. This is the reason for further NaCl deposition on top of areas 1 and 2. After the 90 s roughening that occurs for area 3, the NaCl shutter is closed,

RHEED moved to area 4, and GaAs deposition initialized. The RHEED stays in each of the following 3 locations for 1 min before moving to the next without any pause in GaAs deposition.

S6. Deposition of Ge on NaCl thin films and RHEED effects

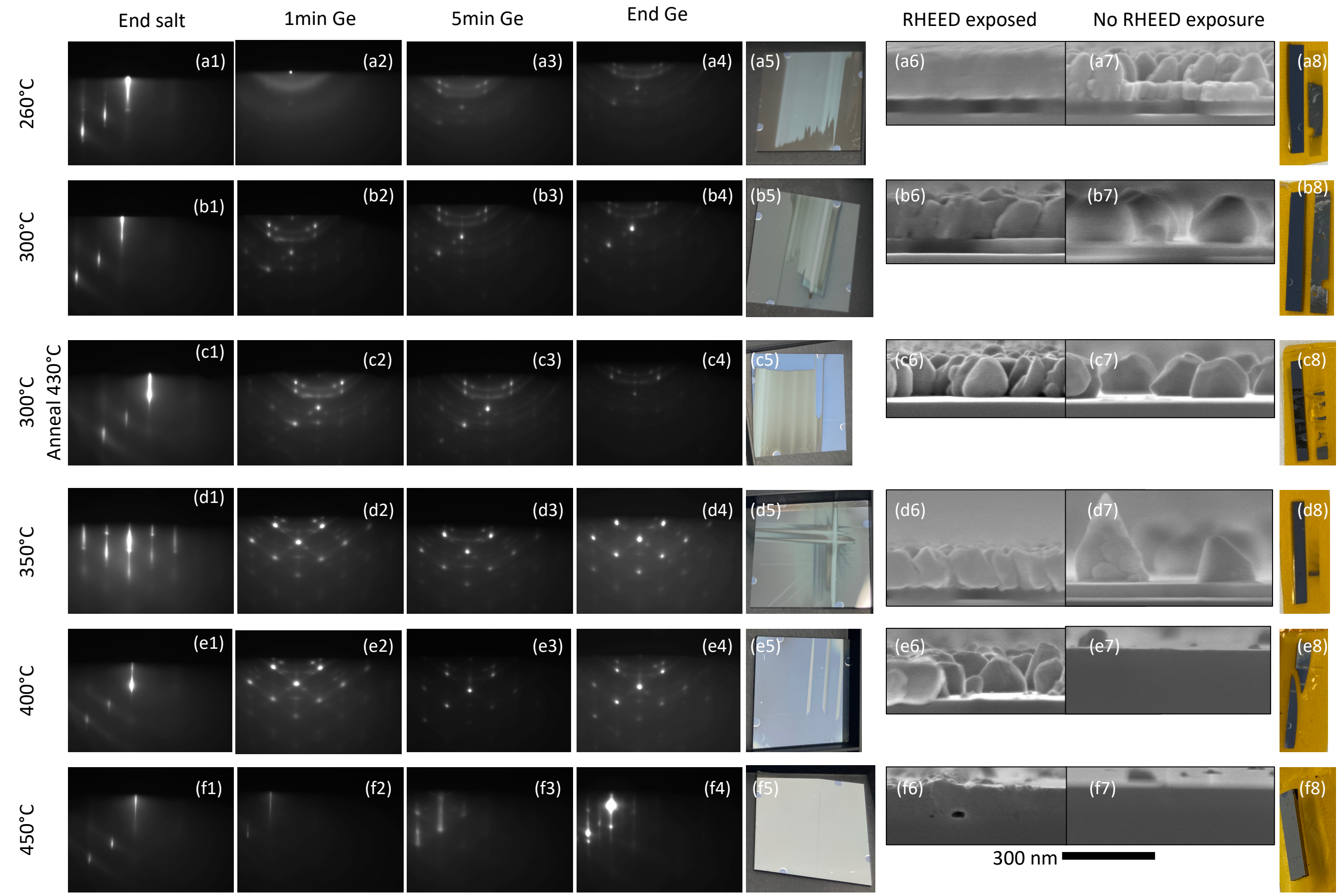

Figure S6: 20 minute Ge depositions on NaCl films on GaAs substrates with deposition beginning at 150°C and continuously until the Ge nucleation is started. (a) ~20min total salt followed by Ge deposition at 260°C, (b) ~23min total salt followed by Ge deposition at 300°C (c) ~23min total salt followed by Ge deposition at 300°C and annealed at 430°C for 5 minutes (d) ~24.5min total salt followed by Ge deposition at 350°C (e) ~49min total salt followed by Ge deposition at 400°C (f) 83 min total salt followed by Ge deposition at 450°C. RHEED images taken at (1) the end of NaCl deposition, just prior to (2) 1 minute into, (3) 5 minitues into, and (4) at the end of Ge deposition. (5) Images of the samples after removal from the chamber showing streaks from the presence of the RHEED beam throughout growth. (6) Ge films attached to Kapton tape after removal from the substrate via placement in water (lower) and the GaAs substrate (upper). Cross-section SEM images of areas (7) with RHEED exposure and (8) without RHEED exposure (scale bar is the same for all images)

The deposition of elemental Ge on NaCl thin films on GaAs substrates was also investigated because Ge has a similar lattice constant to NaCl and GaAs, but since it is only a single atomic species, there are no added complications with stoichiometry, flux-matching, or overpressures of another volatile species which could affect the growth mode. GaAs films have also been demonstrated on NaCl films previously with the use of Ge interlayers.[15,24] A similar growth scheme as outlined in Supplementary S2(b) is used. NaCl was deposited at low temperatures for a t_{NaCl} and continuously for an additional t_{ramp1} before T_{GaAs1} was reached. Then once the temperature stabilized, Ge was deposited for 20 minutes. The sample was cooled immediately after Ge deposition. A series of growths was performed varying the T_{GaAs1} of Ge from 260-450°C and analyzed using RHEED (along a [110] direction) and cross section SEM. The very clear discrepancy in areas with RHEED exposure (WRE) and areas with no RHEED exposure (NRE) in this more simple growth system was where we realized the importance of the presence of an electron beam.

For the sample with T_{GaAs1}=260°C (Figure S6(a)) the NaCl pattern prior to deposition is still bright and streaky. Upon opening the Ge shutter the pattern quickly dims and after 60s (Figure S6(a2)) the pattern shows diffuse rings meaning the film is highly polycrystalline. After 5 minutes, the wide blurry rings begin to give way to more discrete rings and spots (Figure S6(a3)); but with more time the pattern does not change, except for an overall slight dimming. The initial nucleation is likely highly polycrystalline, and at these low temperatures possibly somewhat amorphous as well, and although further deposition seems to suggest a more crystalline layer, the grains are very small and randomly oriented. The 5th column shows images of the sample after removal from vacuum, and here the RHEED exposed area can be seen clearly in the central region. Cross section SEM for areas with RE and with NRE are shown in Figures S6(a6 and a7), respectively. The area WRE shows the darker NaCl film completely covered by a dense Ge film. In the area with NRE there is still a NaCl layer of similar thickness. However, the Ge overlayer, while still mostly covering the NaCl, is very rough with significant variations in height. One thing that was not mentioned in the main text as it is outside the scope of this work is the ability of the NaCl to function as a water-soluble release layer. The final column of this figure (Figures S6(a8-f8) shows a piece of the sample that was cleaved off and had the Ge attached to Kapton tape. The substrate and tape were submerged in water, where the NaCl would rapidly dissolve allowing for near-immediate removal of the Ge overlayer. Figure S6(a8) clearly shows the central (area WRE) lifted off. The edge areas are less dark because the thickness of the Ge is still transparent, but this region also lifted off.

Similar growth and analysis of a sample with T_{GaAs1}=300°C was performed (Figures S6(b)). The starting NaCl is similar to the previous sample, but the initial Ge deposition no longer shows blurry rings. Instead, the pattern is very spotted with more discrete rings. Thus, when grown at this temperature the Ge has a higher degree of short-range ordering. Further growth results in dimming of the rings and the shadow spots, while the primary spots (which would match up with NaCl) become brighter (Figure S6(b4)). This suggests that with further growth a more textured film is being achieved, and with further growth maybe even something approaching single-crystalline could be achieved. Because it would be nucleated on material consisting of multiple orientations, it would likely have a high defect density. Again, looking at the sample after removal from the chamber the area WRE can be clearly differentiated in the center (Figure S6(b5)). Cross section SEM shows that the film in the central area WRE is not quite a cohesive film, and although the thickness is similar to the previous case, there is the appearance of discrete columnar islands. In the area with NRE, the islands are much more discrete and further apart. But again, with

persistent NaCl layers beneath, the tape peeled easily from the substrate either with or without placement in water and the Ge can be seen still stuck to the tape (Figure S6(b8)).

The previous sample is repeated again, except with an additional anneal at 430°C for 5 minutes in an effort to coalesce islands and achieve more crystalline material (Figures S6(c)). Unsurprisingly, the first three RHEED patterns look nearly identical as the growths are identical up until this point. However, the pattern after the annealing (Figure S6(c4)) dimmed considerably, with no apparent reduction in the relative brightness of the rings or shadow spots. Signifying that the crystallinity was not improved and the film just got rougher. The image of the sample after removal from the chamber shows the left half of the sample exposed to RHEED clearly. SEM no longer shows the presence of a NaCl layer in either area. Instead, Ge islands are simply sitting on the GaAs substrate surface. The density of islands in the area WRE is higher than the area with NRE, but because neither area had a conformal film to protect the NaCl for heating to high temperatures, the NaCl desorbs away. When attempting to remove the Ge islands from the substrate (Figure S6(c8)) it was more sporadic. Perhaps some NaCl existed some places that were not imaged in SEM, facilitating removal, while in other regions the Ge fused to the substrate and could not be lifted off.

Another sample was grown, now increasing T_{GaAs1} to 350°C (Figures S6(d)). The RHEED pattern of the NaCl is again bright and streaky. But after the first minute of Ge deposition, the pattern is slightly different than previous cases. There are not full rings, rather they are broken into spots, often intersecting the symmetric shadow spots about the primary. The primary spots are very bright and show chevrons connecting them. This indicates a more textured film than the previous cases. With further growth, the primary spots get relatively brighter compared to the rings and shadow spots as well signifying an improvement of the crystal structure. The lines from the RHEED exposure can be seen again, including marks from when the sample was rotated 90° during growth (Figure S6(d5)). The SEM images still show persistent NaCl layers in both areas WRE and NRE (Figures S6(d6,7)). In the area WRE the Ge more closely resembles a film of approximately the target thickness, while outside of the RHEED-exposed area the density of the islands is much lower. These discrete islands also can be much taller than expected and are extremely faceted. This suggests that the Ge adatoms can move around on the NaCl surface until they find an existing Ge island to help grow. When attempting to remove the overlayer from the substrate as shown in Figure S6(d8), the tape peeled easily from the substrate. There is a clear line where the area was exposed to the RHEED. Moving away from that, the dark color fades. This is because where the RHEED was is the dense film, and moving away from this region the density of the Ge islands decreases, until they are no longer visible with a camera.

The RHEED patterns when increasing T_{GaAs1} to 400°C (Figures S6(e1-4)) are similar to the previous case. And while streaks can be seen on the sample from the presence of the RHEED beam (Figure S6(e5)), the SEM images reveal discrete islands (Figure S6(e6)), and nothing resembling a full film. The nucleation rate of Ge on NaCl at these temperatures must be lower, and the NaCl is definitely desorbing faster. Even with a longer initial NaCl deposition (t_{NaCl}) of 30 min, there is no NaCl present underneath these Ge islands. Additionally, in the area with NRE, neither a NaCl layer nor Ge islands are really observed. It is likely that in this area the nucleation density of Ge is so low that the NaCl fully desorbs and heteroepitaxial Ge is deposited directly on the GaAs. Attaching the sample to tape was not able to remove any material that was visible to the eye.

A final sample with t_{NaCl}=60 minutes was done using a T_{GaAs1}=450°C (Figures S6(f)). The RHEED pattern of the NaCl layer at this temperature is still streaky but starting to blur, which is understandable because the substrate temperature is almost equivalent to the effusion cell temperature. After closing the NaCl and opening the Ge for 60 seconds, the pattern has dimmed further as the sample was roughening as Ge was going down on an actively desorbing surface. With continued growth, the pattern begins to reveal spotty streaks and eventually finishes with a bright pattern that has rotational symmetry (Figure S6(f4)). However, unlike the previous samples the area WRE is barely discernable in Figure S6(f5). Cross section SEM reveals why; in the area WRE it appears only slightly rougher than the area with NRE. Small voids are present under the surface at a depth approximately equal to the target Ge layer. This would suggest that the area with NRE (Figure S6(f7)) is heteroepitaxial Ge on GaAs, and that the layer on top of the voids in the area WRE is also Ge that formed only fast enough for some NaCl to sublime out leaving behind a pore. Attempted removal of material from this substrate (Figure S6(f8)) unsurprisingly yielded no results.

S7. Details of low temperature As-adsorption with RHEED images

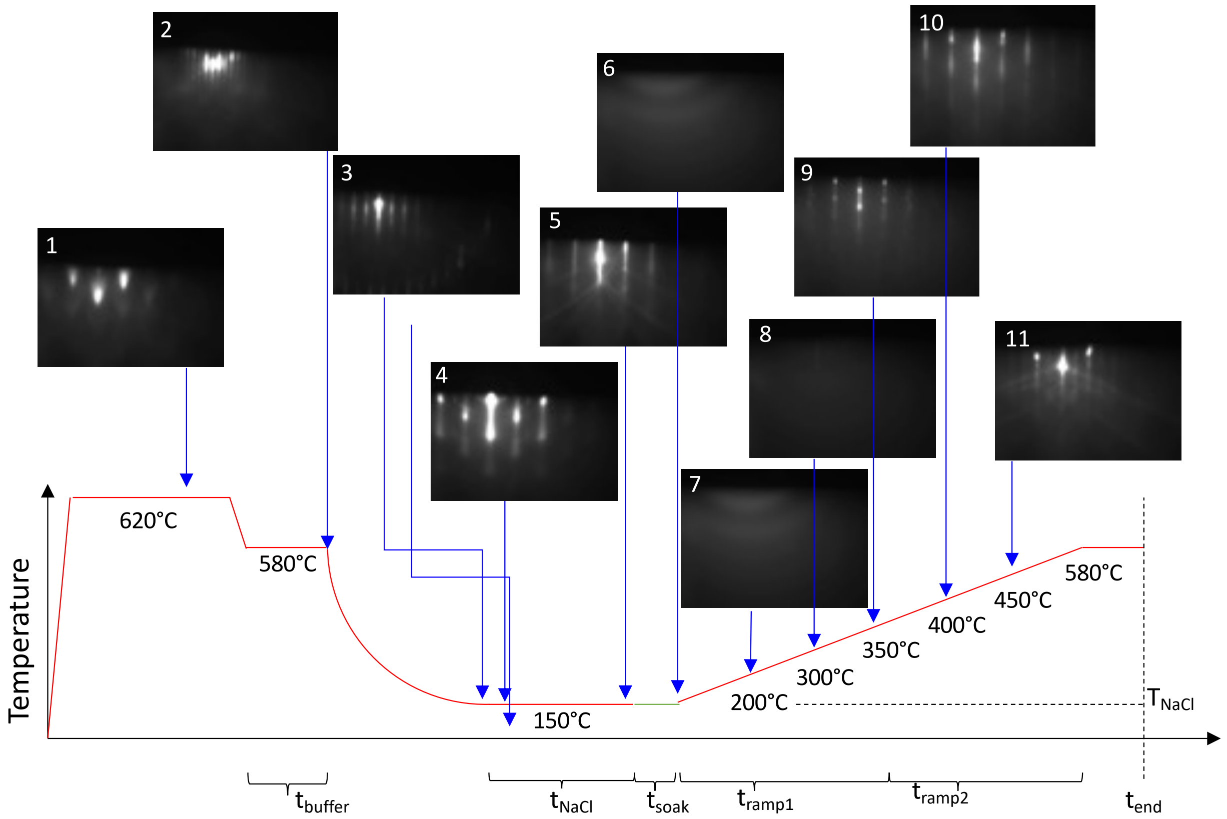

Figure S7: Growth schematic and RHEED images at different points throughout the growth of a sample using RHEED-assisted As-adsorption

This section pertains to the growths in section 2.3.2 in the main text and will give discussion around the electron beam induced As-adsorption. A representative growth schematic with RHEED images at different points throughout the growth labeled with numbers 1-11. The first three steps are identical for all other growths in this study, moving sequentially through the growth, we begin with a 25 minute oxide desorb and the first 3 steps are the same

1. After a few minutes at 620°C under a high As background pressure (6.9×10^{-6} torr), the GaAs surface which was once covered with an oxide and did not give a good RHEED pattern, now shows a bright pattern typical of an oxide free surface. The sample is cooled to 580°C and a 300 nm GaAs buffer is grown to clean up the surface.
2. The RHEED now shows a bright streaky 2×4 pattern (shown here along the $<\bar{1}10>$) at the end of the buffer layer signifying a smooth, well-ordered and As-stabilized surface. The sample is kept under a lower As background pressure ($\sim1.0\times10^{-6}$ torr) while cooling until ~340°C before closing the As and cooling fully to T_{NaCl}.
3. A c(4×4) reconstruction appears with cooling under a constant As-flux at intermediate temperatures and persists down to T_{NaCl} without losing any broadening of dimming of the peaks.

The following two images are of NaCl deposition at T_{NaCl}=150°C, however the trends observed are relatively similar for other T_{NaCl} shown in the main text of this study. More details on the NaCl deposition can be found elsewhere.[25]

4. Upon opening the NaCl shutter the GaAs reconstruction disappears quickly, and the pattern is replaced by spotty streaks. There are also faint rings present during the early stages. This suggests that during the formation of the first few MLs of deposition the NaCl is not perfectly smooth and has some degree of polycrystallinity.
5. With further NaCl deposition the streaks become tighter and Kikuchi lines become visible. The surface becomes smoother and appears single crystalline with the spacing between streaks similar to that of the original GaAs.

The following steps pertain only to growths with the RHEED induced As-adsorption.

6. The NaCl shutter is closed and the temperature is held constant. An As flux= 1.22×10^{15} atoms/cm^2/s (matching that of the Ga-flux used for 33 nm/min deposition rates) is supplied to the surface. Under constant RHEED exposure, the pattern transitions over the course of a few seconds from streaky (5) to diffuse (6) suggesting the condensation of amorphous material. However, when the location of the RHEED beam is moved pattern 5 (bare NaCl) is again observed, but it immediately begins to fade until a pattern matching (6) is observed. This stepping of the RHEED beam (which is ~1mm wide) is repeated until the amount of desired area is covered and appears diffuse. Examples of the stark differences caused in areas scanned by the RHEED can be seen in the images of 2×2 cm samples pictured in Supplementary sections 6 and 8.
7. Once the desired amount of surface is exposed to the RHEED beam, the sample is heated (at a rate of 20°C/min) under the same As-flux. The pattern remains unchanged with heating to 200°C (7).
8. Heating further to 300°C, a very faint primary streak starts to appear.
9. Once the temperature reaches 350°C (9) the pattern reveals obvious spotty streaks that match perfectly with the NaCl. Excess As desorbs from GaAs at ~320°C, thus we believe the same thing is happening with material on a NaCl surface. However, one must be careful because any additional exposure of the RHEED beam at temperatures <320°C can re-condense amorphous material, leading to the false assumption that there is still amorphous material everywhere, when in reality areas that were not re-exposed have fully desorbed all condensed material and would already display a pattern similar to (9).
10. By the time the sample is heated to 400°C the spots present at lower temperatures have transitioned into a brighter, streakier pattern. Even though NaCl adsorption is high at these temperatures, cross-section SEM images of samples where GaAs deposition begins at this temperature (Figure 8 main text) reveals persistent NaCl layers. Thus, this pattern is representative of a NaCl surface.
11. Further heating to 450°C shows the brightening of the primary spot, and the reappearance of reconstructions. Because of the reintroduction of the surface reconstructions, we believe that in this pattern is now representative of the GaAs surface. It is worth noting that the t_{NaCl} for this growth is 10 minutes, and the NaCl thickness is only ~30 nm and does not take long to desorb. Cross section SEM in this case shows only smooth GaAs and no evidence that a NaCl layer was ever deposited.

    If t_{NaCl} is increased to 60 minutes (~180 nm) the RHEED pattern does not yet show reconstructions at this point because not all of the NaCl has desorbed. Despite the RHEED

signifying the presence of NaCl at the onset of nucleation at 450°C in this case, there is no observable NaCl layer via SEM measurements. Instead, small holes are observed at the interface. Thus, because the NaCl is still so volatile, it fully desorbs prior to coalescence of a full GaAs film to protect it.

S8. Quantitative pole figures

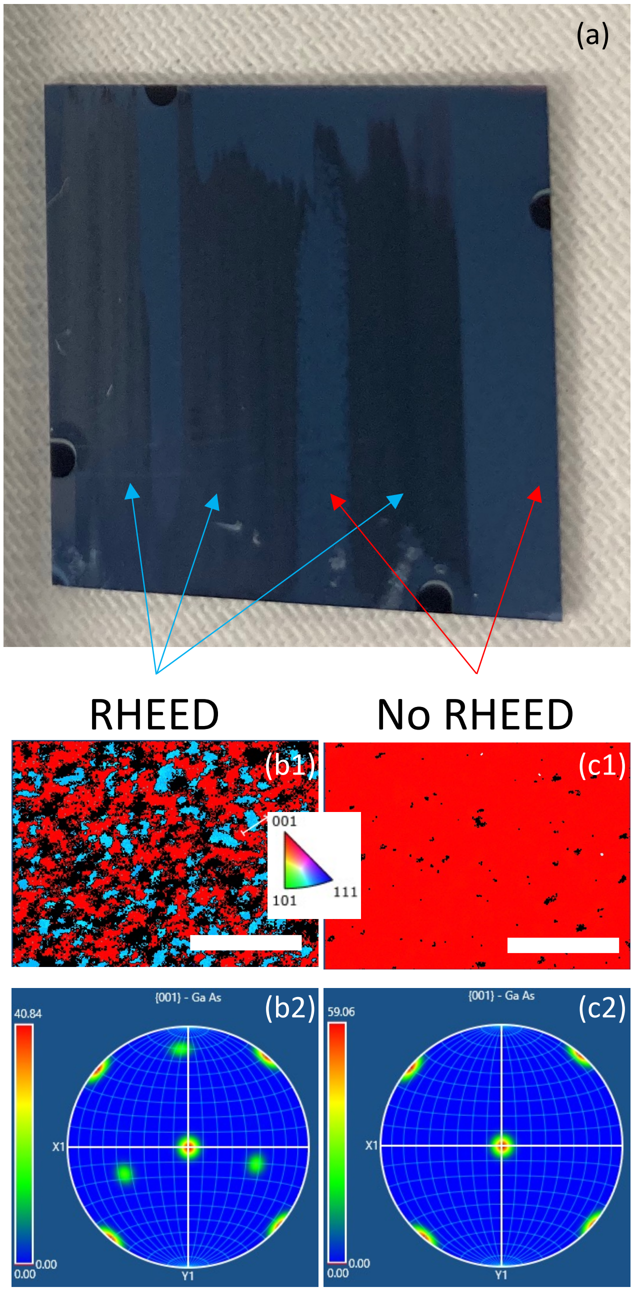

Figure S8: (a) Image of sample from Figure 8 in the main manuscript with (1) EBSD orientation maps and (2) the corresponding pole figure for the (b) RHEED exposed areas and (c) areas without RHEED exposure.

This section serves to highlight and support the data contained in Figure 8 in the main text. Figure S8(a) shows an image of the sample after removal from the vacuum chamber. When inside the chamber, the RHEED beam was incoming from the direction perpendicular to the top edge of the sample. The area exposed to the RHEED appears darker marked by the light blue lines. The red lines point out areas that were not exposed to RHEED, either deliberately or because the

glancing angle beam was blocked by the sample holder clips or flakes on the sample holder. In the absence of any flakes present on the holder (as was observed with other samples), the beam can be scanned across the entirety of the sample during the As-adsorb step to provide a relatively uniform large area. The presence of a shadow from the sample holder clip is not avoided with a single scan, but if the sample were rotated 90° and the beam moved across again it could cover that area. This was not attempted.

Figures S8(b1,c1) are copied from the main text, but now displayed with their respective quantitative pole figures (PFs). Both PFs show the angle of {100} planes with respect to the surface normal. The central spot (at 0°) is indicative of the red areas present in the electron back scatter diffraction (EBSD) orientation maps. The additional set of 4 spots located at 90° from the center, and ~45° from the X and Y axes correspond to the other planes of a single (001) oriented crystal. The 45° misorientation is because the samples are mounted with the cleaved edge ({110}) parallel to the sample holder edge (which is used as the x-y reference). A completely red ((001) up) oriented surface could also be observed with each grain being azimuthaly misoriented (fiber oriented). In a case such as this a full ring would be observed around the outside if there was zero rotational registry with the underlying material, or multiple sets of spots could be observed around the edge if specific rotations were preferred. Here, only a single set of 4-fold rotationally symmetric spots are observed, and because the samples are 0±0.1° offcut, we can say these grains are exactly aligned with the substrate.

As discussed in the main text, the RHEED exposed area in Figure S8(b2) shows another color (light blue) corresponding to {221} grains. The PF of this region shows a single set of spots correspnding to only a single rotational domain for these grains. The origin of this orientation, why there is only a single rotation, and how to remove them to move toward more single-crystal material is the subject of separate work.

References

(15) Sharma, S.; Favela, C. A.; Sun, S.; Selvamanickam, V. Novel Epitaxial Lift-Off for Flexible, Inexpensive GaAs Solar Cells. *Conference Record of the IEEE Photovoltaic Specialists Conference* **2020**, *2020-June*, 0744–0746. https://doi.org/10.1109/PVSC45281.2020.9300363.

(24) Shuskus, A. J.; Cowher, M. E. *Fabrication of Monocrystalline GaAs Solar Cells Utilizing NaCl Sacrificial Substrates*; 1984. https://doi.org/https://www.osti.gov/biblio/6317217.

(25) May, B. J.; Kim, J. J.; Walker, P.; Moutinho, H. R.; McMahon, W. E.; Ptak, A. J.; Young, D. L. Molecular Beam Epitaxy of GaAs Templates on Water Soluble NaCl Thin Films. *Journal of Crystal Growth* **2022**, *586*, 12667.

(26) Tang, F.; Parker, T.; Wang, G. C.; Lu, T. M. Surface Texture Evolution of Polycrystalline and Nanostructured Films: RHEED Surface Pole Figure Analysis. *Journal of Physics D: Applied Physics* **2007**, *40* (23). https://doi.org/10.1088/0022-3727/40/23/R01.
